# Octopamine increases the excitability of neurons in the snail feeding system by modulation of inward sodium current but not outward potassium currents

**DOI:** 10.1186/1471-2202-6-70

**Published:** 2005-12-06

**Authors:** Ágnes Vehovszky, Henriette Szabó, Christopher JH Elliott

**Affiliations:** 1 Balaton Limnological Research Institute, Hungarian Academy of Sciences, PO Box 35, Tihany, H-8237 Hungary.; 2 Department of Biology, University of York, PO Box 373, York, YO1 5YW, UK.

## Abstract

**Background:**

Although octopamine has long been known to have major roles as both transmitter and modulator in arthropods, it has only recently been shown to be functionally important in molluscs, playing a role as a neurotransmitter in the feeding network of the snail *Lymnaea stagnalis*. The synaptic potentials cannot explain all the effects of octopamine-containing neurons on the feeding network, and here we test the hypothesis that octopamine is also a neuromodulator.

**Results:**

The excitability of the B1 and B4 motoneurons in the buccal ganglia to depolarising current clamp pulses is significantly (P << 0.05) increased by (10 μM) octopamine, whereas the B2 motoneuron becomes significantly less excitable. The ionic currents evoked by voltage steps were recorded using 2-electrode voltage clamp. The outward current of B1, B2 and B4 motoneurons had two components, a transient *I*_A _current and a sustained *I*_K _delayed-rectifier current, but neither was modulated by octopamine in any of these three buccal neurons. The fast inward current was eliminated in sodium – free saline and so is likely to be carried by sodium ions. 10 μM octopamine enhanced this current by 33 and 45% in the B1 and B4 motoneurons respectively (P << 0.05), but a small reduction was seen in the B2 neuron. A Hodgkin-Huxley style simulation of the B1 motoneuron confirms that a 33% increase in the fast inward current by octopamine increases the excitability markedly.

**Conclusion:**

We conclude that octopamine is also a neuromodulator in snails, changing the excitability of the buccal neurons. This is supported by the close relationship from the voltage clamp data, through the quantitative simulation, to the action potential threshold, changing the properties of neurons in a rhythmic network. The increase in inward sodium current provides an explanation for the polycyclic modulation of the feeding system by the octopamine-containing interneurons, making feeding easier to initiate and making the feeding bursts more intense.

## Background

Molluscan feeding, with its repetitive protraction and retraction of the radula to ingest food, has provided a simple model system for the study of central pattern generator neuronal networks and of the way in which the pattern is reshaped by neuromodulators [1,2]. In the buccal ganglia of the pond snail, *Lymnaea stagnalis*, octopamine is released by 3 octopamine-immunoreactive (called OC) interneurons [3] onto all the identified neurons (both interneurons and motoneurons) in the buccal feeding system. The effects of octopamine are particularly interesting because it has a double role: both short-term ( = neurotransmitter) and long-term ( = neuromodulator). The short term effects of the OC cells are to excite protraction phase neurons (e.g. B1 motoneurons), inhibit retraction phase neurons (e.g. B2, B3 motoneurons), and make electrical connections with the neurons which fire in the same phase (swallowing) of feeding cycle as the OC interneurons (e.g. B4 motoneurons) [4]. However, the synaptic outputs of the OC interneurons do not account for all the changes that these interneurons have on the feeding pattern: they also produce a long lasting (polycyclic) modulation of the feeding system. At the network level, the OC interneurons make the protraction phase modulatory interneurons (SO and N1L) much more effective in driving fictive feeding, making the feeding pattern stronger and more robust. This effect can occur even if the OC interneuron is stimulated 6 s (the length of two feeding cycles) before the protraction phase neurons [5].

The network effects of the OC neurons can be accounted for by two cellular processes. First, they produce an increase in the excitability of the protraction phase interneurons (SO, N1L), so that the same current pulse gives more action potentials. Secondly, they increase the strength of the synapses made between protraction phase neurons, including the synapses from the SO and N1L to the N1M CPG interneuron and B1 motoneuron. Both these effects are mimicked by bath-applied octopamine in the range 1–10 μM, which is below the threshold at which octopamine directly depolarises the membrane potential [4].

An increase in membrane excitability has been found to underlie many behavioural processes, including contributions to the modulation seen during learning [6,7] and that seen in crustacean pattern generating networks [8]. In these systems, a variety of ionic mechanisms have been found to be substrates of the increase in excitability [9-11]. We now show that two of the three largest buccal motoneurons, B1 and B4 are more excitable in the presence of octopamine. In order to explore the changes in ionic currents by which the OC interneurons produce a change in excitability, we have voltage clamped these cells and examined the action of their transmitter, octopamine on their membrane currents. As a control, we also voltage clamped the B2 motoneuron which shows a decrease in excitability with octopamine. We have modelled the currents seen in the B1 with a Hodgkin-Huxley style simulation. We find that an increase in inward sodium current, of the same magnitude as that recorded in the voltage clamp, produces a lower threshold and an increase in excitability of the B1 motoneuron. An outline of this work, giving initial data, was recently published [12]

## Results

### Octopamine increases the excitability of the B1 and B4, but not B2 motoneuron

**Figure 1:**
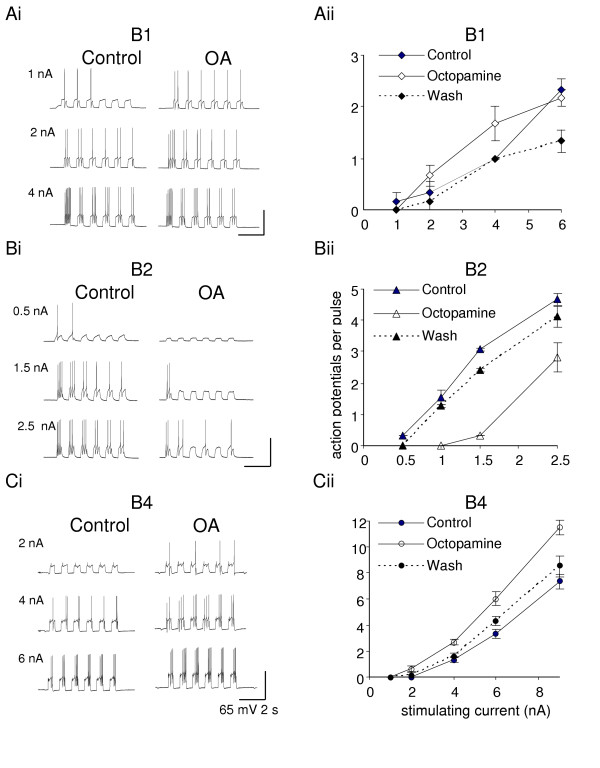
Octopamine (OA, 10 μM) modulates the excitability of the feeding buccal neurons. In the B1 and B4 motoneurons the threshold for action potential generation decreases (A, C), while the B2 motoneuron is less excitable with octopamine in the bath. For the B1 motoneuron the increase of the excitability with octopamine is particularly visible around threshold, while in B4 motoneurons increase of excitability is clear throughout the whole range of the injected current. All control experiments were in Hi-Di saline (to which octopamine was added), and a second electrode was used to inject the constant current pulses to evoke bursts of action potentials. Summary graphs represent dose-response relationship: injected current values (X) versus the mean (± SE) number of action potentials (Y) per 0.5 s depolarizing pulse. *N *= 6 (Aii), *N *= 3 (Bii), *N *= 6 (Cii).

Excitability experiments were performed in Hi-Di to decrease spontaneous synaptic inputs on the cell under study. This made the neurons silent and only the intracellular pulses were able to evoke action potentials. In Hi-Di saline, a series of depolarising pulses of increasing amplitude was used to determine the threshold and excitability of the B1, B2 and B4 motoneurons (Fig. 1). The averaged data show that the mean thresholds are just under 1 and 0.5 nA (B1, B2) and between 2 – 4 nA (B4). Above threshold the number of action potentials increases with stimulus current, though the B1 curve starts to flatten off when six times the threshold current is applied, indicating a sigmoidal stimulus/response curve. (For the B2 and B4 cells, our data only cover the lower part of the curve.)

In 10 μM octopamine, the threshold of the B1 and B4 motoneuron is reduced while the B2 threshold is increased. For supra-threshold currents, the B1 and B4 motoneuron generate more action potentials in octopamine than control saline, while B2 generates less. With two to four times threshold stimulus current, these differences are significant (comparison of individual pairs of experimental recordings by the Wilcoxon test: B1 P <= 0.008; B2 P <= 0.008; B4 P < 0.002). With high stimulus currents, the sigmoidal curves for control and octopamine treatments start to merge together. The effect of octopamine on excitability is at least partially reversed by washing for 10 minutes.

### Outward currents are not affected by octopamine

**Figure 2:**
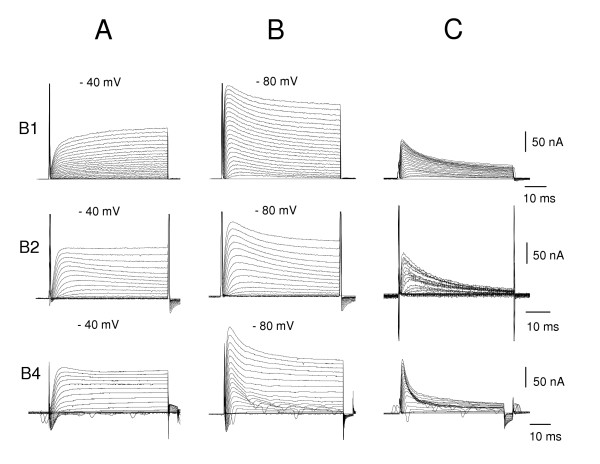
A series of voltage-activated current traces of B1, B2, B4 motoneurons evoked by 5 mV depolarizing voltage steps between -60 mV and +40 mV. A. From a holding potential of -40 mV, initial fast inward current (seen on B2 and B4) is followed by a slowly activating outward current (B1, B2, B4 cells) which does not inactivate during the 50 ms current step. B. The same voltage protocol starting from -80 mV holding potential evokes an additional, fast outward current component on all three motoneurons. C. Subtracting the two current records shows the initial transient outward current is a fast inactivating outward current during the course of the 50 ms voltage steps. All experiments in normal saline.

When any of the three large buccal neurons, B1, B2 or B4 are held in voltage clamp at -40 mV in normal saline, depolarizing pulses elicit an outward current (Fig. 2A). This outward current reaches its peak within 15 ms and is thereafter maintained for the duration of the 50 ms pulse. (In most recordings from the B4 neuron, the smooth outward current traces are interrupted by small spikes. These occur when the large neurons in the B4 cluster, which is electrically coupled to the B4 neuron, fire action potentials.)

**Figure 3:**
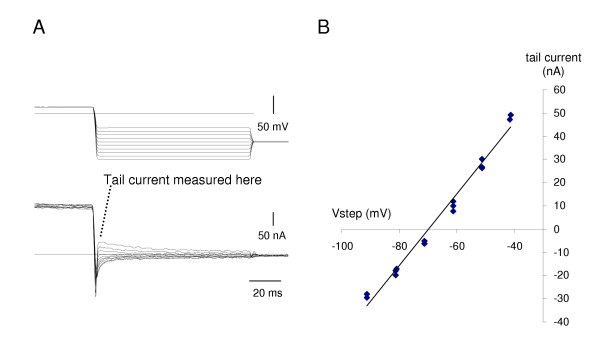
Reversal of the outward current in the B1 motoneuron. A. Following a 400 ms step to +15 mV, activating the outward current, the voltage was stepped down and the amplitude of the tail current measured immediately upon settling. Upper trace: voltage clamp output, lower trace current. B. The tail current is approximately linear with voltage between -80 and -40 mV, with some rectification, and reverses at -70 mV. In 4 replicate preparations, the mean reversal potential was -67 ± 2.6 mV.

**Figure 4:**
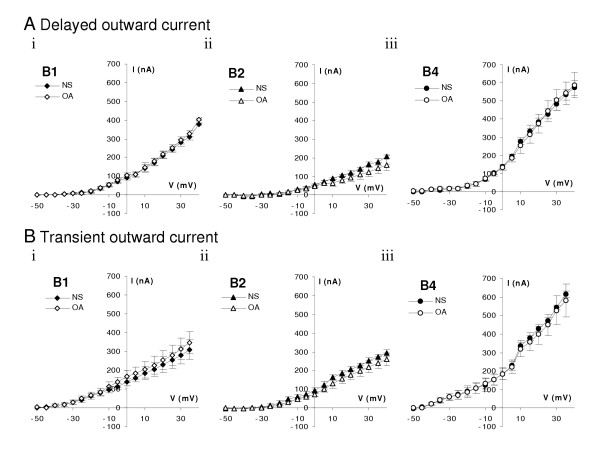
The outward components of the voltage evoked currents are not affected by octopamine. A. Current-voltage relationship of the peak values of the delayed outward currents recorded on B1 (*N *= 8), B2 (*N *= 6) and B4 (*N *= 8) neurons do not show any significant change in the presence of 10 μM octopamine. B. Peak values of the transient outward current component overlap suggesting no significant change of these currents when octopamine (10 μM) is added to the bath, B1 (*N *= 6), B2 (*N *= 6) and B4 (*N *= 8). Normal saline: filled symbols; octopamine: empty symbols. Mean ± SE of currents at end of a 50 ms pulse as in Fig. 2.

The sustained outward current activates at about -30 mV, and increases as the step becomes more positive (Fig. 4A). In the B1 neuron, its reversal potential is -67 ± 2.6 mV (mean ± SE, N = 4) (Fig. 3). This was determined by activating the outward current with a step to +15 mV, followed by clamping at values between -120 and -40 mV and measuring the tail currents. Although well fitted by a straight line, some rectification is observed. The sustained outward current is blocked by tetraethylammonium (TEA) (Fig. 7A), like the delayed rectifier, *I*_*KV *_seen in other molluscan neurons [13]. The kinetics of the outward current, like that of *Anisodoris *neurons [14], is not well fitted by a single exponential raised to a power between 1 and 4. While Connor & Stevens' data [14,15] was fitted with the product of two terms, we achieved a better fit using the sum of two processes, suggesting that the B1 cell may have multiple outward currents. The more rapid component (NA in our worksheet) corresponds with a steeply voltage-activated potassium current, while the slower process (NB) may represent one of the other potassium currents, e.g. a calcium- activated potassium current [13,16,17].

**Figure 5:**
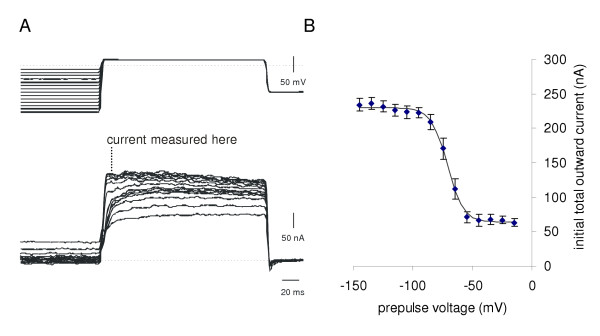
Inactivation of the transient outward current in the B1 motoneuron. A. Holding the voltage for 800 ms at different values, and stepping to a fixed potential of +15 mV, determines the inactivation of the transient outward current. The transient outward current was measured at its peak value. B. Summary data for 11 runs from 4 preparations, showing mean ± SE total outward current. The solid line shows a fit of the Boltzmann equation to the mean data, with a half voltage (*h*) of -71 mV and slope (*s*) of -6.6 mV.

When the B1, B2 and B4 neurons are stepped from a more negative holding potential, -80 mV, the outward current shows an initial peak that is not seen at -40 mV (Fig. 2B). The steady-state current is often the same as that reached when the holding potential was -40 mV. Subtraction of the curves shows a rapidly activating, quickly inactivating outward current (Fig. 2C). This current reaches its peak after 3–6 ms and declines to 50% within 15–20 ms in the B1 and B2 motoneurons, while the half-life is less than 10 ms in the B4 motoneuron. It activates at -50 mV and increases as the potential becomes more positive (Fig. 4B). It is blocked by 4-aminopyridine (4-AP) but not by TEA (Fig. 7A, B). In these characteristics, it resembles the *I*_*A *_currents first recorded in molluscan neurons by Connor & Stevens [15] and the two A currents reported by Alekseev and Ziskin [18]. Over the first 50 ms, the rise and fall of the *I*_*A *_current is well fitted with the product of a single exponential, raised to the power 4 (rising), and a single exponential (decaying). A linear approximation to the time constant gave a good fit over the range -30 to +30 mV. For the inactivation of *I*_*A*_, we did not use the *Lymnaea *data of Alekseev & Zaykin [19], because its half-inactivation at -97 mV predicts that holding at -80 mV should only give a small *I*_*A*_, whereas we found that holding at -80 mV gave a substantial *I*_*A *_(Fig 2). We therefore measured the inactivation of *I*_*A *_in the B1 motoneuron, giving a half-inactivation value of -71 mV (Fig. 5), closer to the -74 mV determined by Connor & Stevens [14].

The summary current-voltage relationship (Fig. 4A, B) shows that the peak outward currents are largest in the B4 neuron and smallest in the B2 neuron. The ratio of *I*_*A *_to *I*_*KV *_(*I*_*A *_/ *I*_*KV*_), measured from recordings of steps to 0 mV, is larger (1.7) in B2 than in B1 or B4 (both 1.4).

Application of octopamine has no significant effect on the amplitude or activation voltage of either the delayed rectifier or transient components of the outward current in any of the B1, B2 or B4 neurons (Fig. 4A, B.

### Fast Inward current increased by octopamine in B1 and B4 but not B2 neurons

**Figure 6:**
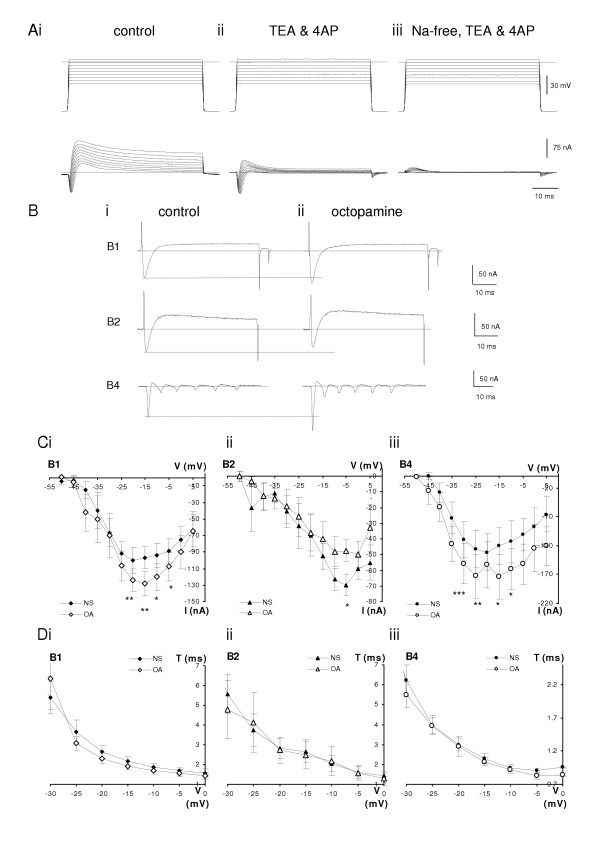
Analysis of the inward currents of buccal neurons. Ai. A series of voltage-steps from a holding potential of -80 mV to between -35 mV and +5 mV on B1 motoneuron. In normal saline an initial fast inward current is followed by outward current. Aii. In saline containing 50 mM tetraethylammonium (TEA) and 4 mM 4-aminopyridine (4-AP) the inward current component is slightly increased while both (the transient and delayed) outward current strongly inhibited. Aiii Changing to Na free saline with the same TEA and 4-AP concentrations shows only a small transient outward current while the inward current is blocked completely. B. Addition of octopamine to normal saline enhances the amplitude of the fast inward currents in B1 and B4 neurons but not in the B2 motoneuron. Individual current traces of representative experiments from -50 mV stepped to -20 mV, -10 mV and -20 mV, respectively in normal saline. The fast inward current is followed by a slower outward component (Bi). In the presence of 10 μM octopamine the fast inward components are increased on B1 and B4 neurons without substantial changes of the outward currents, while the inward current on B2 neuron is decreased (Bii). C. I-V characteristics of the voltage-evoked fast inward currents show increased peak values of the B1 (Ci, n = 8) and B4 (Ciii, n = 8) neurons in the presence of 10 μM octopamine. B2 neurons (n = 6) show decreased amplitudes of the fast inward current (Cii). D. The time at which the peak inward current was observed is not affected by 10 μM octopamine in the B1, B2 or B4 neurons (same preparations as C). In B, C and D experiments were done in normal saline (NS filled symbols) or normal saline supplemented with 10 μM octopamine (OA empty symbols). Mean ± SE. Asterisks mark significance levels of 5% (*), 1%(**) and 0.1% (***).

From a holding potential of -50 mV, the voltage clamp traces show clear transient inward currents in normal saline in each of the B1, B2 and B4 neurons (Fig. 6A, B). The inward currents last less than 10 ms before they are overtaken by outward currents. The inward current activates with the first step to -45 mV (Fig. 6C), and with the next few steps, the amplitude increases while the latency to the peak current decreases. In the B1 and B4 neurons, the peak inward current is reached when the step is to between -25 and -15 mV. We conclude from these properties that this current is a Na^+ ^current like those in other molluscan preparations [14]. This was confirmed by a series of saline replacement experiments. The current was not reduced when TEA or 4-AP was applied; instead it was slightly increased (Fig. 6Aiii), When sodium was removed from the saline (Fig. 6 Aii), the inward current disappears completely.

The inward current transient was well fitted by the product of a rising exponential raised to the third power times a decaying exponential. The inward current activates and inactivates more quickly than reported in *Anisodoris *[20] or *Aplysia *[9], with lower minimum value and more negative voltage at which the time constant is half maximal (Fig. 6C, D) compared with other *Lymnaea *cells [21]).

In the B2 neuron, the maximum inward current is reached at -10 to -5 mV, and this suggests that the B2 inward current is different from the inward current measured on B1 and B4.

Bath applied octopamine, 10 μM, increases the size of the fast inward current in the B1 and B4 neurons, but no increase is seen in the B2 neuron (Fig. 6). This is clear from the individual steps (Fig. 6B) where the size of the control inward current spike is shown by the dotted line: the B1 and B4 neurons respond to octopamine with a larger inward current. This is also reflected in the summarised current-voltage graphs (Fig. 6C). For the B1 neuron, the peak inward current is seen with steps to -15 mV and octopamine increases this from -96 ± 13 nA to -128 ± 15 nA (a 33% increase). Paired t-tests show that the increase in fast inward current is significant for steps in the range -20 mV to -5 mV (*P *<< 5%). For B4, the inward current at a step to -15 mV increases from -121 ± 32 to -173 ± 39 nA (45%) and the paired t-tests indicate a significant increase over most of the steps to -30 to -20 mV. The time for the inward current to reach its peak is not altered by the application of octopamine (Fig. 6D).

In contrast, for the B2, the inward current in octopamine is not increased; rather it is less than in the control. At the peak current, seen with steps to -5 mV, the current in octopamine is 73% of the control; over the range -25 to +5 mV the inward current in octopamine is 77% of the control (Fig. 6Cii). However, only at the steps to -5 and +5 mV is the reduction statistically significant (P < 0.05).

**Figure 7:**
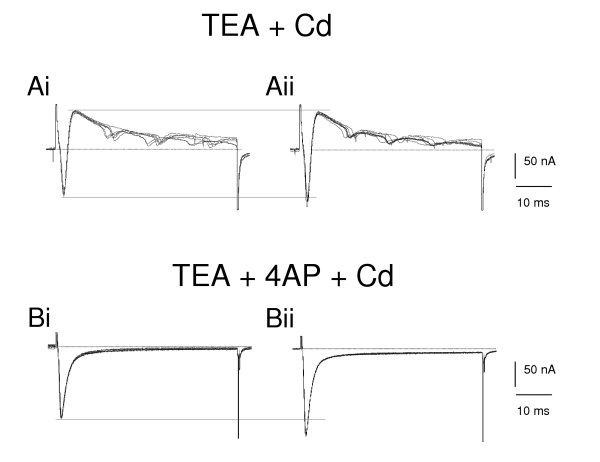
Octopamine selectively enhances the pharmacologically separated fast inward current on B4 neuron. Ai. In the saline containing 50 mM tetraethylammonium (TEA) and 50 μM cadmium, the maintained outward current is reduced and the trace is dominated by a fast inward and transient outward current during five replicate depolarising voltage steps from -80 to -10 mV. (The small inward currents are caused by incomplete voltage clamp because of the high spontaneous activity of electrically coupled B4 cluster neurons). Aii. After 10 μM octopamine is added to this saline the fast inward component is increased without changing the outward component. Bi. In medium containing 50 mM TEA, 4 mM 4-aminopyridine (4-AP) and 50 μM cadmium,, and stepping from -50 to -20 mV, the outward components disappear leaving the fast inward component current (10 replicates). Bii. After adding 10 μM octopamine to the saline, the fast inward current is increased, and a smaller increase is seen in a sustained inward current.

In molluscan neurons, the inward current may be carried not only by sodium but also by calcium ions [22]. In the experiments shown in Fig. 7, we used salines containing 50 μM cadmium to block the calcium component. In the presence of 50 mM tetraethylammonium (TEA), the delayed outward current is blocked (Fig. 7Ai). Addition of 10 μM octopamine to this saline still increases the peak inward current, but leaves the size of the outward current unchanged (Fig. 7Aii). In another preparation, we blocked both transient and delayed outward currents, and any calcium current by using 50 mM TEA, 4 mM 4-AP and 50 μM cadmium (Fig. 7Bi). Addition of 10 μM octopamine to this saline still increases the fast inward current, confirming that the main effect of octopamine is on the fast inward Na^+ ^current (Fig. 7Bii). In this saline the increase is 20%, in the same range as seen in normal saline for the -20 mV voltage step (23 ± 7%).

### Basic physiology of the B1 motoneuron

The resting potential of the B1 motoneuron was 55 ± 1.7 mV (mean ± standard error, SE, N= 16 preparations). Action potentials had a threshold of 1.76 ± 0.23 nA (mean ± SE; N = 15). The mean (± SE) time constant for hyperpolarising pulses was 54 ± 5 ms (N = 11 trials in 7 preparations).

### Simulation

**Figure 8:**
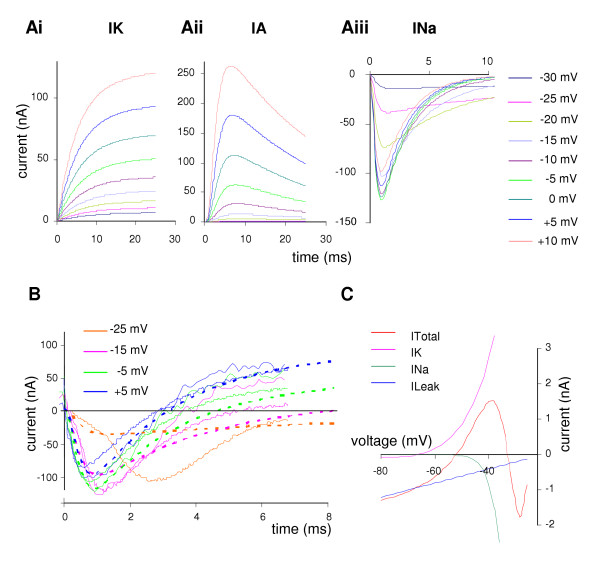
Simulation of ionic currents under voltage clamp in the B1 motoneuron. A. Transient currents from a holding voltage of -80 mV, showing a series of steps starting at -30 mV and increasing in 5 mV increments. Ai. Sustained outward current, the sum of two (increasing) components; compare the B1 data from Fig. 2A. Aii. Transient outward current, rapidly activating and then decaying; compare to data from Fig. 2C. Aiii. Inward current, showing more rapid activation and inactivation as the steps increase; compare to B1 data in Fig. 6. B. Total current in simulation (bold dotted line) from -50 mV to -25 mV, -15 mV, -5 mV and +5 mV, compared with current recordings from 2 different preparations (thin solid lines). At -25 mV the real data show the effects of an action potential propagated from the contralateral cell, starting about 1.5 ms into the recording (so only one preparation is shown at this step). The bold line in black shows the zero current level. C. Simulated Current – Voltage plot, showing the steady-state outward current (IK) the inward current (INa) the leak current (ILeak) and total current (ITotal). The steady state value of the transient outward current is too small to show on this scale. The steady state total current crosses the x-axis at -52 mV with a positive slope, giving this as the calculated stable resting potential.

In voltage clamp simulations, the membrane potential was stepped from a holding voltage of -80 mV to -30 mV, and the simulation repeated, incrementing the step potential by 5 mV. The calculated sustained and transient outward currents (Fig. 8Ai, Aii) closely resemble the forms of the observed currents (Fig. 2A, C). Over the range -30 to +10 mV, the sustained outward current tracks the IV curve (Fig. 4, normal saline) within one standard error at zero mV. The calculated *I*_*A *_current increases more slowly below 0 mV than the observed data, but becomes larger than the observed current thereafter. Simulations run from -40 mV (instead of -80 mV) show the *I*_*A *_reduced from 260 to 3 nA peak for the step to +10 mV. Under voltage clamp, the calculated inward current (Fig. 8Aiii) is transient, with both the onset and decay becoming faster as the steps become more positive.

We have compared the calculated voltage clamp response with that from two representative preparations which had a holding potential of -80 mV (Fig. 8B). With a step to -25 mV, the calculated and actual currents agree well for the first 1.5 ms, but thereafter the actual inward current is much larger. The sudden inflection in the trace suggests that this is the result of an action potential in the (unclamped) contralateral B1 motoneuron. With steps to -15 to +5 mV the agreement between calculated and actual traces is similar to the variation between preparations. There is a good match for both the time of peak inward current, and also for the zero crossing mark. At 6 ms, the total current for the -15 mV and -5 mV is slightly lower than expected, but at +5 mV the calculated trace falls between the two observed traces.

Solving the current clamp equations for the resting potential gave a stable value of -52.5 mV (Fig. 8C). In the region -80 to -60 mV, the maintained outward current, *I*_*K*_, and the leak current dominate. The resting conductance of 88 nS, is the same as the value measured in seven preparations using a series of 6–8 hyperpolarising pulses 1 to 3 nA, 1 to 3 s in duration, which gave a mean B1 conductance of 87 ± 12 nS (equivalent to 13.7 MΩ). The capacitance of the cell was set at 3.5 pF (estimate from hyperpolarising pulses: 4.2 ± 0.4 pF).

**Figure 9:**
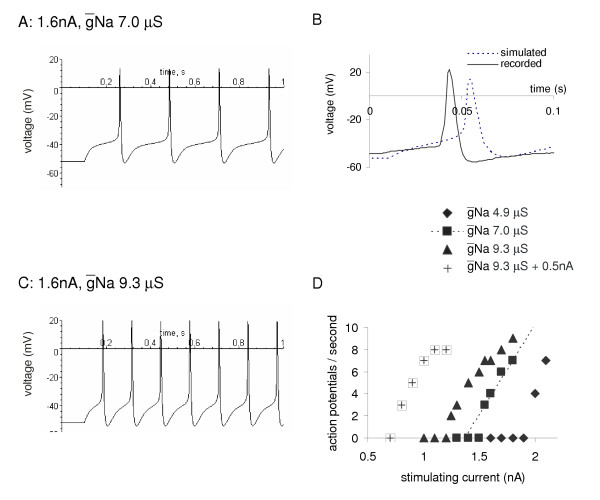
Simulation of constant current injection into the B1 motoneuron in normal saline and with octopamine. A. A current pulse of 1.6 nA with g¯
 MathType@MTEF@5@5@+=feaafiart1ev1aaatCvAUfKttLearuWrP9MDH5MBPbIqV92AaeXatLxBI9gBaebbnrfifHhDYfgasaacH8akY=wiFfYdH8Gipec8Eeeu0xXdbba9frFj0=OqFfea0dXdd9vqai=hGuQ8kuc9pgc9s8qqaq=dirpe0xb9q8qiLsFr0=vr0=vr0dc8meaabaqaciGacaGaaeqabaqabeGadaaakeaacuqGNbWzgaqeaaaa@2E1B@Na at 7 μS, the normal values, depolarises the B1 motoneuron from its resting value of -52 mV and generates four action potentials during the 1 s of simulation. B. Comparison of the action potential generated by simulation with one recorded in a constant current experiment in normal saline (initial resting potential -48 mV, slightly depolarised compared to the mean B1 resting potential, -55 mV). C. The same current pulse as A, but with g¯
 MathType@MTEF@5@5@+=feaafiart1ev1aaatCvAUfKttLearuWrP9MDH5MBPbIqV92AaeXatLxBI9gBaebbnrfifHhDYfgasaacH8akY=wiFfYdH8Gipec8Eeeu0xXdbba9frFj0=OqFfea0dXdd9vqai=hGuQ8kuc9pgc9s8qqaq=dirpe0xb9q8qiLsFr0=vr0=vr0dc8meaabaqaciGacaGaaeqabaqabeGadaaakeaacuqGNbWzgaqeaaaa@2E1B@Na increased by 33% to simulate the effect of 10 μM octopamine and generates 7 action potentials. D. A plot of the firing rate of the simulated B1 motoneuron with different parameters reflecting octopamine action. The squares and dotted line represent the firing rate of the normal model. The triangles show the effect of increasing the maximum sodium conductance (g¯
 MathType@MTEF@5@5@+=feaafiart1ev1aaatCvAUfKttLearuWrP9MDH5MBPbIqV92AaeXatLxBI9gBaebbnrfifHhDYfgasaacH8akY=wiFfYdH8Gipec8Eeeu0xXdbba9frFj0=OqFfea0dXdd9vqai=hGuQ8kuc9pgc9s8qqaq=dirpe0xb9q8qiLsFr0=vr0=vr0dc8meaabaqaciGacaGaaeqabaqabeGadaaakeaacuqGNbWzgaqeaaaa@2E1B@Na) by 33% simulating 10 μM octopamine, reducing the threshold and increasing the firing rate. The crosses show the effect of modelling the increased maximum sodium conductance plus a 0.5 nA octopamine current to simulate the depolarising effect of a higher concentration of octopamine, 100–200 μM, [39]. A further increase in excitability is seen. Decreasing the maximum sodium conductance reduces the number of action potentials (diamonds). Compare these figures with the data shown in Fig. 1A.

In current clamp simulations, positive currents led to depolarisation of the cell membrane. Above 1.55 nA action potentials were produced 25 ms in duration, rising to +20 mV, with a noticeable after-hyperpolarisation (Fig. 9A). Comparison of the shape with recorded action potentials indicates the model is good, though the peak depolarisation is not quite matched. The 1.55 nA threshold is within 1 standard error of that recorded in normal saline (1.76 ± 0.23 nA). Increasing the stimulus current increases the number of action potentials seen in each 1 s of simulation (squares and dotted line in Fig. 9C).

To mimic the effect of 10 μM octopamine, the simulation was run with the maximal sodium conductance (g¯
 MathType@MTEF@5@5@+=feaafiart1ev1aaatCvAUfKttLearuWrP9MDH5MBPbIqV92AaeXatLxBI9gBaebbnrfifHhDYfgasaacH8akY=wiFfYdH8Gipec8Eeeu0xXdbba9frFj0=OqFfea0dXdd9vqai=hGuQ8kuc9pgc9s8qqaq=dirpe0xb9q8qiLsFr0=vr0=vr0dc8meaabaqaciGacaGaaeqabaqabeGadaaakeaacuqGNbWzgaqeaaaa@2E1B@Na) increased by 33% (from 7.0 to 9.3 μS), and this reduces the threshold to 1.25 nA. For each stimulus current, the simulation now produces more action potentials (Fig. 9C), e.g. at 1.6 nA the action potentials increase from 4 to 7 in the 500 ms run (Fig. 9A, B). Reducing the inward conductance to 70% of its control value, to 4.9 μS, raised the threshold and made the firing rate of the simulated cell slower.

In the snail feeding system, prolonged depolarisation may occur through the tonic release of octopamine, serotonin, peptides or other neuromodulators. Although this might be expected to increase the firing of the cell, this is not always the case. In Hodgkin-Huxley simulations, addition of steady depolarising currents can lead to a reduction in activity, because the sodium channels become inactivated. We therefore modelled the effect of a continuous 0.5 nA depolarising current into the B1 on the number of action potentials produced by sharp current pulses. This changes the resting potential to -47 mV; it also lowers the threshold and increases the firing rate (Fig. 9C).

We also tested the effects of increasing the sodium channel conductance in two publicly available models of other molluscan neurons. The simplest model is the space-clamped Hodgkin-Huxley model of a squid axon which has only a single inward and single outward current. Running the implementation by Bezanilla [23] the threshold to a 30 ms stimulus is reduced from 0.47 to 0.3 μA when the maximum sodium conductance (g¯
 MathType@MTEF@5@5@+=feaafiart1ev1aaatCvAUfKttLearuWrP9MDH5MBPbIqV92AaeXatLxBI9gBaebbnrfifHhDYfgasaacH8akY=wiFfYdH8Gipec8Eeeu0xXdbba9frFj0=OqFfea0dXdd9vqai=hGuQ8kuc9pgc9s8qqaq=dirpe0xb9q8qiLsFr0=vr0=vr0dc8meaabaqaciGacaGaaeqabaqabeGadaaakeaacuqGNbWzgaqeaaaa@2E1B@Na) is increased by 33%. This also increases the excitability from 2 to 3 action potentials in the 30 ms, 2 μA pulse. We chose a model of the abdominal sensory neuron [9] because this models a range of calcium and potassium currents and the calcium levels inside the cell and therefore treats more factors than our Maple simulation. This is a good choice from the range of published SNNAP models because it seems possible that some of our potassium current may be calcium dependent. Starting from the implementation downloaded in the SNNAP package, increasing g¯
 MathType@MTEF@5@5@+=feaafiart1ev1aaatCvAUfKttLearuWrP9MDH5MBPbIqV92AaeXatLxBI9gBaebbnrfifHhDYfgasaacH8akY=wiFfYdH8Gipec8Eeeu0xXdbba9frFj0=OqFfea0dXdd9vqai=hGuQ8kuc9pgc9s8qqaq=dirpe0xb9q8qiLsFr0=vr0=vr0dc8meaabaqaciGacaGaaeqabaqabeGadaaakeaacuqGNbWzgaqeaaaa@2E1B@Na by 33% decreased the threshold from 0.6 to 0.54 nA (a 10% reduction). It also increased the excitability, for example with a 1.6 nA stimulus, the number of action potentials in a 2 s pulse increased from 2 to 3.

## Discussion

### Octopamine enhances the excitability of B1 and B4 motoneurons

We have extended our previous observations that low concentrations of octopamine increase the excitability of buccal feeding interneurons [24] to show that octopamine has the same effect on the B1 and B4 motoneurons. Their action potential threshold is reduced, and supra-threshold stimuli evoke more action potentials in octopamine than normal saline. This is a specific cellular response, not some general effect, because the B2 motoneuron becomes less, not more, excitable. The behavioural consequence of this increase in excitability will be to make the motoneurons that drive feeding movement (protraction phase – B1, retraction phase B4) more responsive to CPG input. Their additional action potentials will serve to make the contractions of the muscles they innervate (B1: salivary duct; B4 the *anterior jugalis *muscle of the buccal mass) more powerful. The effects on excitability work in conjunction with the synaptic effects, since the octopamine-containing (OC) interneuron chemically excites B1, and has an electrical synapse with B4 [4]. The next sections of the *Discussion *address the cellular and ionic mechanism of this increase in excitability.

### Overview of the voltage activated currents in B1, B2 and B4

The ionic currents in large molluscan neurons have been extensively analysed ever since the 2 microelectrode voltage clamp technique was introduced in the 1960s [25]. Although the network connections of the *Lymnaea *buccal neurons have been extensively analysed [1,26], their ionic currents have not been described previously. Each of the B1, B2 and B4 motoneurons shows a transient and a delayed outward potassium current and an inward sodium current. These currents are similar to the accumulated data from other neurons in *Aplysia, Helix *and *Lymnaea *[9,16,27,28].

### Outward currents

Both transient (*I*_*A*_) and delayed outward potassium currents (*I*_*KV*_) are present in all three buccal motoneurons (B1, B2 and B4). *I*_*A *_and *I*_*KV *_activate at -45 and -30 mV respectively and are blocked separately by 4-AP and TEA. The transient current is inactivated at a holding potential of -40 mV. The delayed potassium current has two kinetic components, one fast and steeply voltage dependent, and a slower one, which may be due to a calcium-activated potassium current. This would fit with both the kinetics and with the I-V curve from three neurons. In these, the IV curve was extended to +120 mV (data not shown) and it sagged above +80 mV. A 30 ms prepulse to 0 mV abolishes the sag, and this suggests a role for a calcium-activated potassium current [16].

Each cell type has its own typical shape to its outward current trace. The most characteristic difference is that the transient current in the B4 motoneuron inactivates more quickly than that of the B1 or B2 neurons, and this may indicate differences in the *I*_*A *_subtypes expressed in the buccal motoneurons [18]. Secondly, the magnitude of the current differs between cells, with the B4 neuron having more current in steps to, for example, 0 mV, than B1 or B2. As the B4 neuron is smaller than B1, this means that the channel density must be higher in B4. Thirdly, the shape of the outward current trace reflects the ratio of *I*_*A *_/ *I*_*KV *_and this is larger in B2 than B4, implying that expression of the channels are controlled independently.

We have no data suggesting the presence of two other common channel types, either S-channels or inward rectifiers, though Straub & Benjamin [29] had predicted an *I*_*h *_current in B4 from current clamp recordings. Hyperpolarizing steps to -100 to -150 mV showed no activating currents in any of the B1, B2 or B4 motoneurons under our conditions.

### Inward currents

An inward transient sodium current (*I*_*Na*_), is the main inward current in the B1 and B4 cells, with the peak current seen in steps to -25 to -15 mV. However, the fast inward current in the B2 neuron differs, with the peak current occurring at less negative potentials, (-10 to -5 mV). This suggests that the B2 inward current may be a mixture of *I*_*Na *_and a high-voltage activated (HVA) Ca^++ ^current. HVA Ca^++ ^currents have been reported from isolated buccal neurons [30] and from other *Lymnaea *neurons, including the CGC [27] and caudo-dorsal cells [31].

### Effect of octopamine

At 10 μM, octopamine does not affect the resting membrane potential of the buccal neurons B1 and B4 although this concentration is enough to modulate their excitability. 10 μM octopamine is also enough to produce significant changes in the ionic currents evoked by voltage steps in the B1 and B4 motoneurons.

In the B1 and B4 neurons, low concentrations of octopamine, affect the inward, but not the outward currents. The mean value of the inward Na^+ ^current is increased by 33% (B1) – 45% (B4), and the difference is significant at the 0.5% level for one and two data points. The octopamine I-V curve appears as an amplified version of the control, with similar maximal activation voltage. Our simulations show that the effects produced by low concentrations of octopamine on the voltage activated currents will be synergistic to the effects of high (100–500 μM) concentrations of octopamine on membrane potential, a depolarisation of about 10 mV [32]. At the B1 motoneuron, as octopamine arrives, it will increase excitability though an increase in the transient inward current; as the concentration increases, the membrane will depolarise so that the cell is closer to its threshold and fires faster in response to the same input.

In the B2 neuron, 10 μM octopamine has no effect on the voltage-activated outward currents. However, the B2 motoneuron differs from the B1 and B4 neurons, in that low concentrations of octopamine do not increase the fast inward current. In fact, octopamine reduces the fast inward current in the B2 motoneuron, though this is only just significant at the 5% level at two data points.

The effects of high concentrations of octopamine (100 – 500 μM) on the B2 motoneuron are also different to their effect on the B1 and B4 cells [4,32]. High octopamine concentrations hyperpolarize the B2 cell, which will supplement the initial decrease in excitability seen at low concentrations of octopamine.

### Simulation of B1

We have modelled the voltage-activated currents of B1 motoneuron using a Hodgkin-Huxley style simulation, with a view to confirming that the 33% increase in inward sodium current confers an increase in excitability. In this model, we included a sustained outward current (with two kinetically separate components) and a transient outward current as well as the inward current. The architecture of this model resembles those devised by Connor & Stevens [20] and those implemented using SNNAP [9]. As with these models it generates typical voltage clamp currents, a stable resting and action potential, which resemble the B1 data in shape and size. When positive current is applied, the difference between our data (Fig. 1) and the simulation (Fig. 9) is small: with the default parameters the threshold current modelled in *silico *(1.55 nA) is within one standard error of the average recorded *in vivo *(1.7 ± 0.23 nA, mean ± se).

The main purpose of the simulation is to test in a quantitative manner the effects of increasing the inward sodium current on excitability. The simulation clearly shows that a 33% increase the maximum inward sodium reduces the threshold and enhances the firing rate in response to the same constant current stimulus. This increase in excitability when the inward sodium conductance is increased is preserved in our simulation when it is started with different parameters, for example when the maximum sustained outward current is reduced. When a small depolarising current is added to the simulation, a further increase in excitability is seen.

A decrease in threshold and increase in the spikes elicited by constant current pulses when the inward sodium current is increased is also seen in two other molluscan action potential simulations, of a gastropod sensory neuron and of the squid giant axon. Thus an increase in inward sodium current seems to be a general mechanism for an increase in excitability, as it is seen in all 3 models we have used. Conversely, a 33% reduction in the inward sodium conductance reduces excitability and increases the threshold.

### Mechanism(s) for octopamine to increase B1 and B4 excitability

The octopamine – induced increase in the fast inward current means that depolarizing synaptic inputs will open more Na^+ ^channels and so be more likely to generate an action potential in the presence of octopamine. This provides a straightforward explanation of the increase in excitability, which was manifested in B1 and B4 as more action potentials were evoked for the same depolarising stimulus. We have confirmed this effect using in a quantitative simulation. Our observation of octopamine-induced increase in *I*_*Na *_in two cell types suggests that a similar cellular mechanism may underlie the increase of excitability of the SO, N1L and N1M. Our simulation also supports the idea that the decrease in excitability in the B2 motoneuron may result in part from the small decrease in inward current.

### Comparison with other mechanisms to increase excitability

A very wide range of neuromodulators have been shown to control the excitability of neurons in snails, crustacea and vertebrates. Among these, modulation of inward currents has been demonstrated by amines [33] and peptides [34]. Changes in excitability may also arise from modulation of outward currents. In many cases, the behavioural context or endogenous source of the modulator is not clear, but this is not the case with the serotonergic modulation of gill-withdrawal in *Aplysia*. In classical conditioning, reduction in *I*_*KS *_underlies the increase in excitability, but an independent reduction in *I*_*KV *_increases spike width – for review see [6]. In another well-established model, *Hermissenda *type B photoreceptors, an increase in excitability is produced by reduction in *I*_*A *_and the calcium-activated K^+ ^current during conditioning [35].

As well as changes during conditioning, modulation of neuronal excitability plays a fundamental part of the reconfiguration of neuronal networks. Again, in many model systems, control of voltage activated channels occurs in synchrony with effects on resting membrane potential. This has been extensively explored in the stomatogastric ganglia of crustacea [8], but these are hard to put in a behavioural context. Voltage clamp data has been obtained from many neurons in molluscs, but in most cases the functional role is again missing.

## Conclusion

We have shown that low concentrations of octopamine modulate the excitability of motoneurons in the snail feeding system. The changes in the inward sodium current quantitatively account for the increased excitability of the B1 motoneuron. Thus, for the first time in molluscs, we are able to relate voltage clamp data using a quantitative simulation to a rhythmic network where we have a good data on how behavior is changed by the modulatory effect of octopamine [32]. The increase in inward sodium current provides an explanation for the polycyclic modulation of the feeding system by the OC interneurons. A similar effect on the SO and N1L interneurons would well account for neuromodulation by the OC interneurons, making feeding easier to initiate and making the feeding bursts more intense [5,24].

## Methods

### Snails

Pond snails, *Lymnaea stagnalis *were collected from the Kis-Balaton region of Hungary, and maintained in aquaria with flowing filtered Balaton lake water. They were fed *ad libitum *with lettuce.

### Dissection

Experiments were done at room temperature to which the snails had been acclimatised for > 24 hours. The CNS, including the buccal ganglia was dissected free of other tissue and pinned out in a Sylgard dish through which saline could be pumped. The outer (white spotted) layers of connective tissue were removed from the buccal ganglia with forceps, and the inner layers digested for 2–5 minutes with 0.1% Sigma Protease XIV.

### Current clamp

The large buccal neurons were identified visually from previous maps [36] and impaled gently with two electrodes. These were pulled from borosilicate glass, filled with a solution of 4 M potassium acetate and 0.3 M potassium chloride, and were dipped in Rotring Ink P to aid visibility. Their resistance was 12–14 MΩ. One electrode was used to record voltage data, the other to inject current. Recordings were stored using DasyLab (version 5) running on a PC through a National Instruments PC-6035E interface card.

### Voltage clamp

Two-electrode voltage clamp was performed as described for other *Lymnaea *preparations [27], using an AxoClamp 2-A or 2-B amplifier. Electrodes were made as described for current clamp, but the puller settings were adjusted so that the resistance of the electrodes was 3–4 MΩ for current electrodes and 12–14 MΩ for voltage electrodes respectively. An aluminium foil barrier was used to reduce capacitance between the electrodes. The large buccal neurons were identified as above and impaled gently with both electrodes under current-clamp conditions. After switching to voltage clamp mode, the gain was increased to 90–100x while observing a 20 mV depolarizing test pulse. The holding potential and voltage clamp protocols were determined using the Strathclyde Electrophysiological software package, versions 2.26 and 3.5.5, running on PC-compatible computers through National Instruments PC-6035E interface cards. Data were sampled at 0.5 to 3 kHz, using the P/N subtraction protocol, with high frequencies removed in software.

Our voltage clamp analysis is restricted by the way that many buccal neurons are electrically coupled. This includes the B4 neurons, which are coupled, not only to their contralateral partner, but also to the B4 cluster neurons that surround them. This has a major impact under voltage clamp, with traces being contaminated with action potentials from the surrounding cells (e.g. Fig 2 and 4A: bottom rows), and we have therefore not analysed the kinetic data from the B4 neurons nor attempted to simulate their activity. The left-right B1 neurons connect in the buccal commissure [36], well away from the cell body, so that this does not impact on the voltage clamp data any more than the presence of the axonal/dendritic branches. The B2 neurons have not been reported to make electrical synapses, either with each other or other buccal neurons.

### Saline solutions

Under control conditions, standard *Lymnaea *saline was pumped through the bath at about 1 ml/min. Current clamp and control voltage clamp recordings were routinely made in normal *Lymnaea *saline, but the current clamp threshold data (Fig. 1) was obtained in Hi-Di saline which reduces synaptic potentials and rhythmic activity. Once voltage clamp was established in normal saline, potassium, sodium and /or calcium currents were blocked by pumping in a replacement saline. The exact compositions of the salines are given in Table 1.

Once a stable recording had been reached, 10 μM octopamine was added to the saline being pumped into the bath and the measurements repeated.

All chemicals were from Sigma.

### Modelling

A Hodgkin-Huxley simulation of the membrane currents in the B1 motoneuron was implemented in the computer algebra package Maple (version 8) [37] based mainly on the data from our voltage clamp recordings. Maple provides a convenient way to input and display the equations relating voltage to current for both the Boltzmann equations and Ordinary Differential Equations (ODEs), and to plot their algebraic or numerical solutions [38]. Appendix 2 shows the Maple output from one simulation run, where the B1 neuron was stimulated with 1.6 nA current. The Maple worksheets (in MPL and MWS format), and typical output (as PDF) are included as Additional Files. Maple was run on a PC or Sun workstation. Unlike a previous Maple simulation of neuron R15 in *Aplysia *[38], there was no need to call an external C-program to solve the ODEs laid out in the Maple worksheets. The Adams integration routine within Maple (lsode default method) was sufficient to deal with these ODEs, even though they are numerically stiff.

Hodgkin-Huxley simulations assume that the proportion of channels open (through activation and inactivation by gates) follows the Boltzmann distribution, regulated by a first order ODE, and that the current flowing through each kind of channel is given by Ohm's law (see Appendix 1). We used this set of equations in the voltage clamp worksheet. In the current clamp worksheet, another ODE, the Capacitor Equation, determines the voltage change from the total current and the membrane capacitance, is added. Our worksheets follow formulation of these equations laid out by Connor & Stevens [20] and Baxter et al. [9].

Our worksheets contain two outward currents (sustained and transient) and an inward current because our Results (see below, Figs. 2, 3, 4, 5, 6, 7) suggest that these are the major currents in the B1 motoneuron. We also include a small linear leak current.

The parameters for our equations were estimated by extracting the voltage clamp data into Excel (Microsoft, Seattle, USA).

The time constants of the outward currents were estimated by fitting exponential curves (raised to integer powers) to the voltage clamp data using the least squares method and the Solver tool. The Solver tool was also used to fit the Boltzmann models (Equations 1 and 3), to give the voltage dependence of the open probabilities of the channels and time constants. For the outward currents a linear approximation of the Boltzmann equation relating the time constant to membrane potential (Equation 3) was valid over the physiological range of membrane potentials. For the inward sodium current, the time constants of Connor & Stevens [20] provided a starting point, and more realistic values for the B1 motoneuron were estimated iteratively, comparing the output of Maple integration with the total (inward + outward) currents recorded at the same membrane potential.

The leak conductance and membrane capacitance was estimated from fitting a single exponential hyperpolarising -1 to -2 nA current clamp stimuli.

The exact equations used and numerical constants are shown in Appendix 2, copied from Maple rtf format output.

We also tested the effects of numerical changes to the inward sodium current on threshold and firing rate in two other publicly available models: the space-clamped squid giant axon [23] using as control values the web-defaults, and an *Aplysia *abdominal neuron implemented in SNNAP [9], using as initial values those in the downloaded files.

## Appendix 1 Outline of Simulation

Hodgkin-Huxley style simulations are based on the idea that ions cross the membrane by flowing though channels controlled by independent gates. The Boltzmann equation gives the steady state proportion of gates (*A*_*inf*_), which will be open at a particular transmembrane voltage (*V*):

Ainf⁡=11+exp⁡(V−hs)     Equation1
 MathType@MTEF@5@5@+=feaafiart1ev1aaatCvAUfKttLearuWrP9MDH5MBPbIqV92AaeXatLxBI9gBaebbnrfifHhDYfgasaacH8akY=wiFfYdH8Gipec8Eeeu0xXdbba9frFj0=OqFfea0dXdd9vqai=hGuQ8kuc9pgc9s8qqaq=dirpe0xb9q8qiLsFr0=vr0=vr0dc8meaabaqaciGacaGaaeqabaqabeGadaaakeaacqWGbbqqdaWgaaWcbaGagiyAaKMaeiOBa4MaeiOzaygabeaakiabg2da9maalaaabaGaeGymaedabaGaeGymaeJaey4kaSIagiyzauMaeiiEaGNaeiiCaa3aaeWaaeaadaWcaaqaaiabdAfawjabgkHiTiabdIgaObqaaiabdohaZbaaaiaawIcacaGLPaaaaaGaaCzcaiaaxMaatCvAUfeBSjuyZL2yd9gzLbvyNv2CaeHbwvMCKfMBHbaceiGaa8xraiaa=fhacaWF1bGaa8xyaiaa=rhacaWFPbGaa83Baiaa=5gacaWFGaGaa8xmaaaa@5504@

where the parameters *h *and *s *provide the half-activation voltage and an indication of the slope of the sigmoidal curve. The proportion of gates open (*A*) will tend towards this according to an ordinary differential equation (ODE):

dAdt=Ainf⁡−Aτ(V)     Equation2
 MathType@MTEF@5@5@+=feaafiart1ev1aaatCvAUfKttLearuWrP9MDH5MBPbIqV92AaeXatLxBI9gBaebbnrfifHhDYfgasaacH8akY=wiFfYdH8Gipec8Eeeu0xXdbba9frFj0=OqFfea0dXdd9vqai=hGuQ8kuc9pgc9s8qqaq=dirpe0xb9q8qiLsFr0=vr0=vr0dc8meaabaqaciGacaGaaeqabaqabeGadaaakeaadaWcaaqaaiabdsgaKjabdgeabbqaaiabdsgaKjabdsha0baacqGH9aqpdaWcaaqaaiabdgeabnaaBaaaleaacyGGPbqAcqGGUbGBcqGGMbGzaeqaaOGaeyOeI0IaemyqaeeabaGaeqiXdq3aaeWaaeaacqWGwbGvaiaawIcacaGLPaaaaaGaaCzcaiaaxMaatCvAUfeBSjuyZL2yd9gzLbvyNv2CaeHbwvMCKfMBHbaceiGaa8xraiaa=fhacaWF1bGaa8xyaiaa=rhacaWFPbGaa83Baiaa=5gacaWFGaGaa8Nmaaaa@5335@

In this equation, the time-constant, *τ(V)*, is a Boltzmann-like function of the membrane voltage (normally with different *h *and *s*):

τ(V)=τmin⁡+τmax⁡−τmin⁡1+exp⁡(V−hs)     Equation3
 MathType@MTEF@5@5@+=feaafiart1ev1aaatCvAUfKttLearuWrP9MDH5MBPbIqV92AaeXatLxBI9gBaebbnrfifHhDYfgasaacH8akY=wiFfYdH8Gipec8Eeeu0xXdbba9frFj0=OqFfea0dXdd9vqai=hGuQ8kuc9pgc9s8qqaq=dirpe0xb9q8qiLsFr0=vr0=vr0dc8meaabaqaciGacaGaaeqabaqabeGadaaakeaacqaHepaDdaqadaqaaiabdAfawbGaayjkaiaawMcaaiabg2da9iabes8a0naaBaaaleaacyGGTbqBcqGGPbqAcqGGUbGBaeqaaOGaey4kaSYaaSqaaeaacqaHepaDdaWgaaWcbaGagiyBa0MaeiyyaeMaeiiEaGhabeaakiabgkHiTiabes8a0naaBaaaleaacyGGTbqBcqGGPbqAcqGGUbGBaeqaaaGcbaGaeGymaeJaey4kaSIagiyzauMaeiiEaGNaeiiCaa3aaeWaaeaadaWcaaqaaiabdAfawjabgkHiTiabdIgaObqaaiabdohaZbaaaiaawIcacaGLPaaaaaGaaCzcaiaaxMaatCvAUfeBSjuyZL2yd9gzLbvyNv2CaeHbwvMCKfMBHbaceiGaa8xraiaa=fhacaWF1bGaa8xyaiaa=rhacaWFPbGaa83Baiaa=5gacaWFGaGaa83maaaa@676F@

In a neuron, the summed conductance (*g*) of this kind of channel is determined by the maximum conductance (*gMax*) and the proportion of channels open. In the simplest case, a channel with *n *uniform gates would follow:

*g *= gMax* *A*^*n *^   *Equation 4*

while for a channel controlled by multiple types of gates (like the inward sodium or transient outward currents) *gMax *would be multiplied by the product of their open probabilities raised to integer powers. The current flowing through the channel (*I*) is then

*I *= (*V *– *E*)* *g *   *Equation 5*

where *E *is the equilibrium reversal potential for the ion which flows through the channel.

This set of equations, replicated for each kind of channel, is sufficient for a voltage clamp simulation, but to calculate a current clamp response, a further ODE is required, the Capacitor equation, which gives the rate of change of voltage from the capacitance (*C*) and overall current:

dVdt=∑I−Istim−C     Equation6
 MathType@MTEF@5@5@+=feaafiart1ev1aaatCvAUfKttLearuWrP9MDH5MBPbIqV92AaeXatLxBI9gBaebbnrfifHhDYfgasaacH8akY=wiFfYdH8Gipec8Eeeu0xXdbba9frFj0=OqFfea0dXdd9vqai=hGuQ8kuc9pgc9s8qqaq=dirpe0xb9q8qiLsFr0=vr0=vr0dc8meaabaqaciGacaGaaeqabaqabeGadaaakeaadaWcaaqaaiabdsgaKjabdAfawbqaaiabdsgaKjabdsha0baacqGH9aqpdaWcaaqaamaaqaeabaGaemysaKKaeyOeI0IaemysaK0aaSbaaSqaaiabdohaZjabdsha0jabdMgaPjabd2gaTbqabaaabeqab0GaeyyeIuoaaOqaaiabgkHiTiabdoeadbaacaWLjaGaaCzcamXvP5wqSXMqHnxAJn0BKvguHDwzZbqegyvzYrwyUfgaiqGacaWFfbGaa8xCaiaa=vhacaWFHbGaa8hDaiaa=LgacaWFVbGaa8NBaiaa=bcacaWF2aaaaa@548F@

where the overall current is the sum of those flowing through each channel (Σ I) less the stimulus current (*I*_*stim*_).

These equations were implemented in Maple (typical output in Appendix 2). The Additional Files show worksheets (in mws [Maple worksheet] and mpl [Maple input] formats) and further output (in pdf format).

## Appendix 2 Maple Output for simulation of B1

This output shows the exact constants and equations used for the simulation shown in Fig. 9A. In this a zero stimulus current was applied for 0.1 ms followed by a stimulus current of 1.6 nA. The full worksheet is available for download in the additional files.

          Constants

          Stimulus current, in nA, at 0.1 ms goes from zero to 1.6 nA

          60

          *Istim *:= if(*t *< 0.100, 0, 1.600)

          Octopamine ligand gated current

          *I*_*OA *:= 0.000

          Capacitance in microF

*          Cm *:= 0.004

          Equilibrium voltages, mV

          Sodium, Potassium, Leak

          28

          *vNa *:= 35

          *vK *:= -67

          *vLeak *:= -20

          Fixed leak conductance

          *gLeak *:= 0.020

          Sodium current

          maximum conductance, m and h components

          with max value and time constant as functions of voltage

          *gbarNa *:= 7.000

          minf:= v→11+e(−3−1/8 v)
 MathType@MTEF@5@5@+=feaafiart1ev1aaatCvAUfKttLearuWrP9MDH5MBPbIqV92AaeXatLxBI9gBaebbnrfifHhDYfgasaacH8akY=wiFfYdH8Gipec8Eeeu0xXdbba9frFj0=OqFfea0dXdd9vqai=hGuQ8kuc9pgc9s8qqaq=dirpe0xb9q8qiLsFr0=vr0=vr0dc8meaabaqaciGacaGaaeqabaqabeGadaaakeaaieGacqWFTbqBcqWFPbqAcqWFUbGBcqWFMbGzcqWFGaaiieaacqGF6aGocqqG9aqpcqqGGaaicqWG2bGDcqGHsgIRdaWcaaqaaiabigdaXaqaaiabigdaXiabgUcaRmXvP5wqSXMqHnxAJn0BKvguHDwzZbqegyvzYrwyUfgaiqqacaqFLbWaaWbaaSqabeaacqGGOaakcqGHsislcqaIZaWmcqGHsislcqaIXaqmcqGGVaWlcqaI4aaocqqGGaaicqWG2bGDcqGGPaqkaaaaaaaa@5110@

          taum :=v→0.000+112511+e(0.500 v+ 20.000)
 MathType@MTEF@5@5@+=feaafiart1ev1aaatCvAUfKttLearuWrP9MDH5MBPbIqV92AaeXatLxBI9gBaebbnrfifHhDYfgasaacH8akY=wiFfYdH8Gipec8Eeeu0xXdbba9frFj0=OqFfea0dXdd9vqai=hGuQ8kuc9pgc9s8qqaq=dirpe0xb9q8qiLsFr0=vr0=vr0dc8meaabaqaciGacaGaaeqabaqabeGadaaakeaacqWG0baDcqWGHbqycqWG1bqDcqWGTbqBcqqGGaaicqqG6aGocqqG9aqpcqWG2bGDcqGHsgIRcqaIWaamcqGGUaGlcqaIWaamcqaIWaamcqaIWaamcqGHRaWkdaWcaaqaaiabigdaXaqaaiabigdaXiabikdaYiabiwda1aaadaWcaaqaaiabigdaXaqaaiabigdaXiabgUcaRmXvP5wqSXMqHnxAJn0BKvguHDwzZbqegyvzYrwyUfgaiqqacaWFLbWaaWbaaSqabeaacqGGOaakcqaIWaamcqGGUaGlcqaI1aqncqaIWaamcqaIWaamcqqGGaaicqWG2bGDcqGHRaWkcqqGGaaicqqGYaGmcqqGWaamcqqGUaGlcqqGWaamcqqGWaamcqqGWaamcqqGPaqkaaaaaaaa@5FD4@

          hinf :=v→11+e(7.632 + 0.263 v)
 MathType@MTEF@5@5@+=feaafiart1ev1aaatCvAUfKttLearuWrP9MDH5MBPbIqV92AaeXatLxBI9gBaebbnrfifHhDYfgasaacH8akY=wiFfYdH8Gipec8Eeeu0xXdbba9frFj0=OqFfea0dXdd9vqai=hGuQ8kuc9pgc9s8qqaq=dirpe0xb9q8qiLsFr0=vr0=vr0dc8meaabaqaciGacaGaaeqabaqabeGadaaakeaacqWGObaAieGacqWFPbqAcqWFUbGBcqWFMbGzcqqGGaaicqqG6aGocqqG9aqpcqWG2bGDcqGHsgIRdaWcaaqaaiabigdaXaqaaiabigdaXiabgUcaRmXvP5wqSXMqHnxAJn0BKvguHDwzZbqegyvzYrwyUfgaiqqacaGFLbWaaWbaaSqabeaacqqGOaakcqqG3aWncqqGUaGlcqqG2aGncqqGZaWmcqqGYaGmcqqGGaaicqqGRaWkcqqGGaaicqqGWaamcqqGUaGlcqqGYaGmcqqG2aGncqqGZaWmcqqGGaaicqWG2bGDcqGGPaqkaaaaaaaa@5640@

         tauh :=v→0.002+320011+e(0.263 v + 6.395)
 MathType@MTEF@5@5@+=feaafiart1ev1aaatCvAUfKttLearuWrP9MDH5MBPbIqV92AaeXatLxBI9gBaebbnrfifHhDYfgasaacH8akY=wiFfYdH8Gipec8Eeeu0xXdbba9frFj0=OqFfea0dXdd9vqai=hGuQ8kuc9pgc9s8qqaq=dirpe0xb9q8qiLsFr0=vr0=vr0dc8meaabaqaciGacaGaaeqabaqabeGadaaakeaacqWG0baDcqWGHbqycqWG1bqDcqWGObaAcqqGGaaicqqG6aGocqqG9aqpcqWG2bGDcqGHsgIRcqaIWaamcqGGUaGlcqaIWaamcqaIWaamcqaIYaGmcqGHRaWkdaWcaaqaaiabiodaZaqaaiabikdaYiabicdaWiabicdaWaaadaWcaaqaaiabigdaXaqaaiabigdaXiabgUcaRmXvP5wqSXMqHnxAJn0BKvguHDwzZbqegyvzYrwyUfgaiqqacaWFLbWaaWbaaSqabeaacqqGOaakcqqGWaamcqqGUaGlcqqGYaGmcqqG2aGncqqGZaWmcqqGGaaicqWG2bGDcqqGGaaicqqGRaWkcqqGGaaicqqG2aGncqqGUaGlcqqGZaWmcqqG5aqocqqG1aqncqqGPaqkaaaaaaaa@5FB9@

          Sustained Potassium current

          maximum conductance, NA and NB components

          with max value and time constant as functions of voltage

          *gbarKA *:= 1.440

         NAinf :=v→11+e(0.898 − 0.060 v)
 MathType@MTEF@5@5@+=feaafiart1ev1aaatCvAUfKttLearuWrP9MDH5MBPbIqV92AaeXatLxBI9gBaebbnrfifHhDYfgasaacH8akY=wiFfYdH8Gipec8Eeeu0xXdbba9frFj0=OqFfea0dXdd9vqai=hGuQ8kuc9pgc9s8qqaq=dirpe0xb9q8qiLsFr0=vr0=vr0dc8meaabaqaciGacaGaaeqabaqabeGadaaakeaacqWGobGtcqWGbbqqtCvAUfeBSjuyZL2yd9gzLbvyNv2CaeHbwvMCKfMBHbaceiGaa8xAaiaa=5gacaWFMbGaeeiiaaIaeeOoaOJaeeypa0JaemODayNaeyOKH46aaSaaaeaacqaIXaqmaeaacqaIXaqmcqGHRaWkiqqacaGFLbWaaWbaaSqabeaacqGGOaakcqaIWaamcqGGUaGlcqaI4aaocqaI5aqocqaI4aaocqqGGaaicqGHsislcqqGGaaicqqGWaamcqqGUaGlcqqGWaamcqqG2aGncqqGWaamcqqGGaaicqWG2bGDcqGGPaqkaaaaaaaa@5603@

          *tauNA *:= *v *→ 0.038 - 0.000 *v*

          *gbarKB *:= 2.880

          NBinf :=v→11+e(0.589 − 0.068 v)
 MathType@MTEF@5@5@+=feaafiart1ev1aaatCvAUfKttLearuWrP9MDH5MBPbIqV92AaeXatLxBI9gBaebbnrfifHhDYfgasaacH8akY=wiFfYdH8Gipec8Eeeu0xXdbba9frFj0=OqFfea0dXdd9vqai=hGuQ8kuc9pgc9s8qqaq=dirpe0xb9q8qiLsFr0=vr0=vr0dc8meaabaqaciGacaGaaeqabaqabeGadaaakeaacqWGobGtcqWGcbGqtCvAUfeBSjuyZL2yd9gzLbvyNv2CaeHbwvMCKfMBHbaceiGaa8xAaiaa=5gacaWFMbGaeeiiaaIaeeOoaOJaeeypa0JaemODayNaeyOKH46aaSaaaeaacqaIXaqmaeaacqaIXaqmcqGHRaWkiqqacaGFLbWaaWbaaSqabeaacqGGOaakcqaIWaamcqGGUaGlcqaI1aqncqaI4aaocqaI5aqocqqGGaaicqGHsislcqqGGaaicqqGWaamcqqGUaGlcqqGWaamcqqG2aGncqqG4aaocqqGGaaicqWG2bGDcqGGPaqkaaaaaaaa@560F@

          *tauNB *:= *v *→ 0.006 - 0.000 *v*

          Transient Potassium current

          maximum conductance, a and b components

          with max value and time constant as functions of voltage

          *gbarA *:= 12

          ainf :=v→11+e(−0.879 − 0.071 v)
 MathType@MTEF@5@5@+=feaafiart1ev1aaatCvAUfKttLearuWrP9MDH5MBPbIqV92AaeXatLxBI9gBaebbnrfifHhDYfgasaacH8akY=wiFfYdH8Gipec8Eeeu0xXdbba9frFj0=OqFfea0dXdd9vqai=hGuQ8kuc9pgc9s8qqaq=dirpe0xb9q8qiLsFr0=vr0=vr0dc8meaabaqaciGacaGaaeqabaqabeGadaaakeaatCvAUfeBSjuyZL2yd9gzLbvyNv2CaeHbwvMCKfMBHbaceiGaa8xyaiaa=LgacaWFUbGaa8NzaiabbccaGiabbQda6iabb2da9iabdAha2jabgkziUoaalaaabaGaeGymaedabaGaeGymaeJaey4kaScceeGaa4xzamaaCaaaleqabaGaeiikaGIaeyOeI0IaeGimaaJaeiOla4IaeGioaGJaeG4naCJaeGyoaKJaeeiiaaIaeyOeI0IaeeiiaaIaeeimaaJaeeOla4IaeeimaaJaee4naCJaeeymaeJaeeiiaaIaemODayNaeiykaKcaaaaaaaa@55A4@

          *taua *:= *v *→ 0.002 - 0.000 *v*

          binf :=v→11+e(0.152 v + 10.758)
 MathType@MTEF@5@5@+=feaafiart1ev1aaatCvAUfKttLearuWrP9MDH5MBPbIqV92AaeXatLxBI9gBaebbnrfifHhDYfgasaacH8akY=wiFfYdH8Gipec8Eeeu0xXdbba9frFj0=OqFfea0dXdd9vqai=hGuQ8kuc9pgc9s8qqaq=dirpe0xb9q8qiLsFr0=vr0=vr0dc8meaabaqaciGacaGaaeqabaqabeGadaaakeaatCvAUfeBSjuyZL2yd9gzLbvyNv2CaeHbwvMCKfMBHbaceiGaa8Nyaiaa=LgacaWFUbGaa8NzaiabbccaGiabbQda6iabb2da9iabdAha2jabgkziUoaalaaabaGaeGymaedabaGaeGymaeJaey4kaScceeGaa4xzamaaCaaaleqabaGaeiikaGIaeGimaaJaeiOla4IaeGymaeJaeGynauJaeGOmaiJaeeiiaaIaemODayNaeeiiaaIaee4kaSIaeeiiaaIaeeymaeJaeeimaaJaeeOla4Iaee4naCJaeeynauJaeeioaGJaeeykaKcaaaaaaaa@5588@

          *taub *:= *v *→ 0.026 + 0.000 *v*

          Initial conditions, start from equilibrium voltage  (-52.5 mV)

          *v0 *:= -52.500

          Sodium current

          *m0 *:= 0.028

          *h0 *:= 0.998

          Sustained Potassium current

          *NA0 *:= 0.017

          *NB0 *:= 0.015

          Transient Potassium current

          *a0 *:= 0.055

          *b0 *:= 0.057

          Current equations

          Sodium current

          Sustained Potassium current

          Transient Potassium current

          Leak current

          Total ionic current

          *INa *:= 7.000 (v(*t*) - 35) m(*t*)^3 ^h(*t*)

          *IK *:= (v(*t*) + 67) (1.440 NA(*t*)^2 ^+ 2.880 NB(*t*))

          *IA *:= 12 (v(*t*) + 67) a(*t*)^4 ^b(*t*)

          *ILeak *:= 0.020 v(*t*) + 0.0400

          *ITotal *:= 7.000 (v(*t*) - 35) m(*t*)^3 ^h(*t*) + (v(*t*) + 67) (1.440 NA(*t*)^2 ^+ 2.880 NB(*t*))

          +12 (v(*t*) +67) a(*t*)^4 ^b(*t*) + 0.020 v(*t*) + 0.400

          Differential equations

          ODEs for Sodium current

          odem :=ddtm(t)=11+e(−3−1/8 v(t))−m(t)0.000+112511+e(0.500 v(t) + 20.000)
 MathType@MTEF@5@5@+=feaafiart1ev1aaatCvAUfKttLearuWrP9MDH5MBPbIqV92AaeXatLxBI9gBaebbnrfifHhDYfgasaacH8akY=wiFfYdH8Gipec8Eeeu0xXdbba9frFj0=OqFfea0dXdd9vqai=hGuQ8kuc9pgc9s8qqaq=dirpe0xb9q8qiLsFr0=vr0=vr0dc8meaabaqaciGacaGaaeqabaqabeGadaaakeaacqWGVbWBcqWGKbazcqWGLbqzcqWGTbqBcqqGGaaicqqG6aGocqqG9aqpdaWcaaqaaiabdsgaKbqaaiabdsgaKjabdsha0baacqqGTbqBcqqGOaakcqWG0baDcqGGPaqkcqGH9aqpdaWcaaqaamaalaaabaGaeGymaedabaGaeGymaeJaey4kaSYexLMBbXgBcf2CPn2qVrwzqf2zLnharyGvLjhzH5wyaGabbiaa=vgadaahaaWcbeqaaiabcIcaOiabgkHiTiabiodaZiabgkHiTiabigdaXiabc+caViabiIda4iabbccaGiabbAha2jabcIcaOiabdsha0jabcMcaPiabcMcaPaaaaaGccqGHsislcqqGTbqBcqGGOaakcqWG0baDcqGGPaqkaeaacqaIWaamcqGGUaGlcqaIWaamcqaIWaamcqaIWaamcqGHRaWkdaWcaaqaaiabigdaXaqaaiabigdaXiabikdaYiabiwda1aaadaWcaaqaaiabigdaXaqaaiabigdaXiabgUcaRiaa=vgadaahaaWcbeqaaiabcIcaOiabicdaWiabc6caUiabiwda1iabicdaWiabicdaWiabbccaGiabbAha2jabcIcaOiabdsha0jabcMcaPiabbccaGiabbUcaRiabbccaGiabbkdaYiabbcdaWiabb6caUiabbcdaWiabbcdaWiabbcdaWiabbMcaPaaaaaaaaaaa@7FFE@

          odeh :=ddth(t)=11+e(7.632 + 0.263 v(t))−h(t)0.002+320011+e(0.263 v(t) + 6.395)
 MathType@MTEF@5@5@+=feaafiart1ev1aaatCvAUfKttLearuWrP9MDH5MBPbIqV92AaeXatLxBI9gBaebbnrfifHhDYfgasaacH8akY=wiFfYdH8Gipec8Eeeu0xXdbba9frFj0=OqFfea0dXdd9vqai=hGuQ8kuc9pgc9s8qqaq=dirpe0xb9q8qiLsFr0=vr0=vr0dc8meaabaqaciGacaGaaeqabaqabeGadaaakeaacqWGVbWBcqWGKbazcqWGLbqzcqWGObaAcqqGGaaicqqG6aGocqqG9aqpdaWcaaqaaiabdsgaKbqaaiabdsgaKjabdsha0baacqqGObaAcqqGOaakcqWG0baDcqGGPaqkcqGH9aqpdaWcaaqaamaalaaabaGaeGymaedabaGaeGymaeJaey4kaSYexLMBbXgBcf2CPn2qVrwzqf2zLnharyGvLjhzH5wyaGabbiaa=vgadaahaaWcbeqaaiabcIcaOiabiEda3iabc6caUiabiAda2iabiodaZiabikdaYiabbccaGiabbUcaRiabbccaGiabicdaWiabc6caUiabikdaYiabiAda2iabiodaZiabbccaGiabbAha2jabbIcaOiabdsha0jabcMcaPiabcMcaPaaaaaGccqGHsislcqqGObaAcqqGOaakcqWG0baDcqGGPaqkaeaacqaIWaamcqGGUaGlcqaIWaamcqaIWaamcqaIYaGmcqGHRaWkdaWcaaqaaiabiodaZaqaaiabikdaYiabicdaWiabicdaWaaadaWcaaqaaiabigdaXaqaaiabigdaXiabgUcaRiaa=vgadaahaaWcbeqaaiabcIcaOiabicdaWiabc6caUiabikdaYiabiAda2iabiodaZiabbccaGiabbAha2jabcIcaOiabdsha0jabcMcaPiabbccaGiabbUcaRiabbccaGiabbAda2iabb6caUiabbodaZiabbMda5iabbwda1iabbMcaPaaaaaaaaaaa@8564@

          ODEs for Sustained Potassium current

          odeNA :=ddtNA(t)=11+e(0.898 − 0.060 v(t))−NA(t)0.038 − 0.000 v(t)
 MathType@MTEF@5@5@+=feaafiart1ev1aaatCvAUfKttLearuWrP9MDH5MBPbIqV92AaeXatLxBI9gBaebbnrfifHhDYfgasaacH8akY=wiFfYdH8Gipec8Eeeu0xXdbba9frFj0=OqFfea0dXdd9vqai=hGuQ8kuc9pgc9s8qqaq=dirpe0xb9q8qiLsFr0=vr0=vr0dc8meaabaqaciGacaGaaeqabaqabeGadaaakeaacqWGVbWBcqWGKbazcqWGLbqzcqWGobGtcqWGbbqqcqqGGaaicqqG6aGocqqG9aqpdaWcaaqaaiabdsgaKbqaaiabdsgaKjabdsha0baacqqGobGtcqqGbbqqcqGGOaakcqWG0baDcqGGPaqkcqGH9aqpdaWcaaqaamaalaaabaGaeGymaedabaGaeGymaeJaey4kaSYexLMBbXgBcf2CPn2qVrwzqf2zLnharyGvLjhzH5wyaGabbiaa=vgadaahaaWcbeqaaiabcIcaOiabicdaWiabc6caUiabiIda4iabiMda5iabiIda4iabbccaGiabgkHiTiabbccaGiabbcdaWiabb6caUiabbcdaWiabbAda2iabbcdaWiabbccaGiabbAha2jabcIcaOiabdsha0jabcMcaPiabcMcaPaaaaaGccqGHsislcqqGobGtcqqGbbqqcqqGOaakcqWG0baDcqGGPaqkaeaacqaIWaamcqGGUaGlcqaIWaamcqaIZaWmcqaI4aaocqqGGaaicqGHsislcqqGGaaicqqGWaamcqqGUaGlcqqGWaamcqqGWaamcqqGWaamcqqGGaaicqqG2bGDcqqGOaakcqWG0baDcqGGPaqkaaaaaa@78D5@

          odeNB :=ddtNB(t)=11+e(0.589 − 0.068 v(t))−NB(t)0.006 − 0.000 v(t)
 MathType@MTEF@5@5@+=feaafiart1ev1aaatCvAUfKttLearuWrP9MDH5MBPbIqV92AaeXatLxBI9gBaebbnrfifHhDYfgasaacH8akY=wiFfYdH8Gipec8Eeeu0xXdbba9frFj0=OqFfea0dXdd9vqai=hGuQ8kuc9pgc9s8qqaq=dirpe0xb9q8qiLsFr0=vr0=vr0dc8meaabaqaciGacaGaaeqabaqabeGadaaakeaacqWGVbWBcqWGKbazcqWGLbqzcqWGobGtcqWGcbGqcqqGGaaicqqG6aGocqqG9aqpdaWcaaqaaiabdsgaKbqaaiabdsgaKjabdsha0baacqqGobGtcqqGcbGqcqqGOaakcqWG0baDcqGGPaqkcqGH9aqpdaWcaaqaamaalaaabaGaeGymaedabaGaeGymaeJaey4kaSYexLMBbXgBcf2CPn2qVrwzqf2zLnharyGvLjhzH5wyaGabbiaa=vgadaahaaWcbeqaaiabcIcaOiabicdaWiabc6caUiabiwda1iabiIda4iabiMda5iabbccaGiabgkHiTiabbccaGiabbcdaWiabb6caUiabbcdaWiabbAda2iabbIda4iabbccaGiabbAha2jabcIcaOiabdsha0jabcMcaPiabcMcaPaaaaaGccqGHsislcqqGobGtcqqGcbGqcqGGOaakcqWG0baDcqGGPaqkaeaacqaIWaamcqGGUaGlcqaIWaamcqaIWaamcqaI2aGncqqGGaaicqGHsislcqqGGaaicqqGWaamcqqGUaGlcqqGWaamcqqGWaamcqqGWaamcqqGGaaicqqG2bGDcqGGOaakcqWG0baDcqGGPaqkaaaaaa@78DC@

          ODEs for Transient Potassium current

          odea :=ddta(t)=11+e(−0.879 − 0.071 v(t))−a(t)0.002-0.000v(t)
 MathType@MTEF@5@5@+=feaafiart1ev1aaatCvAUfKttLearuWrP9MDH5MBPbIqV92AaeXatLxBI9gBaebbnrfifHhDYfgasaacH8akY=wiFfYdH8Gipec8Eeeu0xXdbba9frFj0=OqFfea0dXdd9vqai=hGuQ8kuc9pgc9s8qqaq=dirpe0xb9q8qiLsFr0=vr0=vr0dc8meaabaqaciGacaGaaeqabaqabeGadaaakeaacqWGVbWBcqWGKbazcqWGLbqzcqWGHbqycqqGGaaicqqG6aGocqqG9aqpdaWcaaqaaiabdsgaKbqaaiabdsgaKjabdsha0baacqqGHbqycqqGOaakcqWG0baDcqGGPaqkcqGH9aqpdaWcaaqaamaalaaabaGaeGymaedabaGaeGymaeJaey4kaSYexLMBbXgBcf2CPn2qVrwzqf2zLnharyGvLjhzH5wyaGabbiaa=vgadaahaaWcbeqaaiabcIcaOiabgkHiTiabicdaWiabc6caUiabiIda4iabiEda3iabiMda5iabbccaGiabgkHiTiabbccaGiabbcdaWiabb6caUiabbcdaWiabbEda3iabbgdaXiabbccaGiabbAha2jabcIcaOiabdsha0jabcMcaPiabcMcaPaaaaaGccqGHsislcqqGHbqycqqGOaakcqWG0baDcqGGPaqkaeaaieaacqGFWaamcqGFUaGlcqGFWaamcqGFWaamcqGFYaGmcqGFTaqlcqGFWaamcqGFUaGlcqGFWaamcqGFWaamcqGFWaamcqGFGaaicqGF2bGDcqGFOaakieGacqqF0baDcqGFPaqkaaaaaa@752F@

          odeb :=ddtb(t)=11+e(0.152 v(t) + 10.758)−b(t)0.026+0.000 v(t)
 MathType@MTEF@5@5@+=feaafiart1ev1aaatCvAUfKttLearuWrP9MDH5MBPbIqV92AaeXatLxBI9gBaebbnrfifHhDYfgasaacH8akY=wiFfYdH8Gipec8Eeeu0xXdbba9frFj0=OqFfea0dXdd9vqai=hGuQ8kuc9pgc9s8qqaq=dirpe0xb9q8qiLsFr0=vr0=vr0dc8meaabaqaciGacaGaaeqabaqabeGadaaakeaacqWGVbWBcqWGKbazcqWGLbqzcqWGIbGycqqGGaaicqqG6aGocqqG9aqpdaWcaaqaaiabdsgaKbqaaiabdsgaKjabdsha0baacqqGIbGycqqGOaakcqWG0baDcqGGPaqkcqGH9aqpdaWcaaqaamaalaaabaGaeGymaedabaGaeGymaeJaey4kaSYexLMBbXgBcf2CPn2qVrwzqf2zLnharyGvLjhzH5wyaGabbiaa=vgadaahaaWcbeqaaiabcIcaOiabicdaWiabc6caUiabigdaXiabiwda1iabikdaYiabbccaGiabbAha2jabbIcaOiabdsha0jabcMcaPiabbccaGiabbUcaRiabbccaGiabbgdaXiabbcdaWiabb6caUiabbEda3iabbwda1iabbIda4iabbMcaPaaaaaGccqGHsislcqqGIbGycqGGOaakcqWG0baDcqGGPaqkaeaacqaIWaamcqGGUaGlcqaIWaamcqaIYaGmcqaI2aGncqGHRaWkcqaIWaamcqGGUaGlcqaIWaamcqaIWaamcqaIWaamcqqGGaaicqqG2bGDcqqGOaakcqWG0baDcqGGPaqkaaaaaa@757F@

          ODE for Voltage as function of current

          Stimulus and octopamine ligand gated currents included here

          *odev *:= ddt
 MathType@MTEF@5@5@+=feaafiart1ev1aaatCvAUfKttLearuWrP9MDH5MBPbIqV92AaeXatLxBI9gBaebbnrfifHhDYfgasaacH8akY=wiFfYdH8Gipec8Eeeu0xXdbba9frFj0=OqFfea0dXdd9vqai=hGuQ8kuc9pgc9s8qqaq=dirpe0xb9q8qiLsFr0=vr0=vr0dc8meaabaqaciGacaGaaeqabaqabeGadaaakeaadaWcaaqaaiabdsgaKbqaaiabdsgaKjabdsha0baaaaa@30D1@ v(*t*) = 285.714 if(*t *< 0.100, 0, 1.600) - 114.286

          - 2000.000 (v(*t*) - 35) m(*t*)^3 ^h(*t*)

          - 285.714 (v(*t*) + 67) (1.440 NA(*t*)^2 ^+ 2.880 NB(*t*))

          - 3428.571 (v(*t*) + 67) a(*t*)^4 ^b(*t*) - 5.714 v(*t*)

          Solve system of ODEs

          *sol100 *:= **proc**(*x*_*lsode*) ... **end proc**

          Plotting...

          Setup plot

          *maxT *:= 1.100

          *mazPT *:= 2000

          "'if(t <. 1,0,1.6)"

          "7.0"

          *sHead *:="Stimulus: 'if(t < 1,0,1.6) gNaMax: 7.0"

          Plotting voltage vs time

          [*odeplot*]

          {Fig. 9A is generated and shown here}

          >

## Authors' contributions

AV and CJHE devised the study. Most voltage clamp experiments were done by AV, with support from HS. CJHE carried out the simulation, and drafted the manuscript. AV revised the text, and all authors contributed to its final version.

## Supplementary Material

Additional File 1 Maple worksheet for the ionic currents in the B1 neuron, including graphs of the time constants and equilibrium proportion of gates open ionic currents against holding voltage. Click here for file

Additional File 2 Maple commands for Additional File 1, for non-GUI interfaces. Click here for file

Additional File 3 Maple output from Additional File 1, captured and converted to PDF. Click here for file

Additional File 4 B1_vc.mws. Maple worksheet for voltage clamp simulation of the B1 neuron, in this case stepping from -50 to -10 mV. Maple users can set v0 and v to the holding and pulse potentials (in mV) and run the simulation. The Maple output includes a current / time plot. Click here for file

Additional File 5 B1_vc.mpl. Maple commands from Additional File 3, for non-GUI interfaces. Click here for file

Additional File 6 B1_time.mws. Maple worksheet for current clamp simulation with the current initially zero and then stepped to 1.6 nA. The output of this worksheet is shown in Appendix 2 and the plot in Fig. 9A. Click here for file

Additional File 7 B1_time.mpl. Maple commands from Additional File 3, for non-GUI interfaces. Click here for file

## Figures and Tables

**Table 1:** Final concentrations (mM) of *Lymnaea *salines

	**Normal saline**	**Hi-Di saline**	**TEA+ 4-AP**	**Na-free TEA + 4-AP**	**TEA + Cd**	**TEA + 4-AP + Cd**
NaCl	24	24	24	--	24	24
KCl	2	2	2	2	2	2
CaCl_2_.2H_2_O	4	14	4	4	4	4
MgCl_2_.6H_2_O	2	8	2	2	2	2
NaH_2_PO_4_.2H_2_O	0.1	0.1	0.1	--	0.1	0.1
NaOH	35	35	35	--	35	35
KOH	--	--	--	40	--	--
HEPES	50	50	50	50	50	50
Tetraethyl-ammonium (TEA) chloride	--	--	50	50	50	50
4 amino-pyridine (4-AP)	--	--	4	4	--	4
N-methyl-D glucamine				50		
CdCl_2_					0.05	0.05

Composition of the *Lymnaea *salines (pH = 7.9).
