# Supplementary material for: Octopamine increases the excitability of neurons in the snail feeding system by modulation of inward sodium current but not outward potassium currents
Source: BMC Neurosci. 2005 Dec 6;6:70. doi: 10.1186/1471-2202-6-70 (PMC1351263; doi:10.1186/1471-2202-6-70)
Supplement: Additional File 3 — Maple output from Additional File 1, captured and converted to PDF. [file 1471-2202-6-70-S3.pdf]

Constants
Equilibrium voltages, mV
Sodium, Potassium, Leak

28

$$vNa := 35$$

$$vK := -67$$

$$vLeak := -20$$

Fixed leak conductance

$$gLeak := 0.020$$

Sodium current
maximum conductance, m and h components
with max value and time constant as functions of voltage

$$gbarNa := 7.000$$

$$minf := v \rightarrow \frac{1}{1 + \mathbf{e}^{(-3 - 1/8\ v)}}$$

$$taum := v \rightarrow 0.000 + \frac{1}{125} \frac{1}{1 + \mathbf{e}^{(0.500\ v + 20.000)}}$$

$$hinf := v \rightarrow \frac{1}{1 + \mathbf{e}^{(7.632 + 0.263\ v)}}$$

$$tauh := v \rightarrow 0.002 + \frac{3}{200} \frac{1}{1 + \mathbf{e}^{(0.263\ v + 6.395)}}$$

Sustained Potassium current
maximum conductance, NA and NB components
with max value and time constant as functions of voltage

$$gbarKA := 1.440$$

$$NAinf := v \rightarrow \frac{1}{1 + \mathbf{e}^{(0.898 - 0.060\ v)}}$$

$$tauNA := v \rightarrow 0.038 - 0.000\ v$$

$$gbarKB := 2.880$$

$$NBinf := v \rightarrow \frac{1}{1 + \mathbf{e}^{(0.589 - 0.068\ v)}}$$

$$tauNB := v \rightarrow 0.006 - 0.000\ v$$

Transient Potassium current
maximum conductance, a and b components
with max value and time constant as functions of voltage

$$gbarA := 12$$

$$ainf := v \rightarrow \frac{1}{1 + \mathbf{e}^{(-0.879 - 0.071\ v)}}$$

$$taua := v \rightarrow 0.002 - 0.000\ v$$

$$binf := v \rightarrow \frac{1}{1 + \mathbf{e}^{(0.152\ v + 10.758)}}$$

$$taub := v \rightarrow 0.026 + 0.000\ v$$

```
Initial conditions, start from equilibrium voltage (-52.5 mV)
```

$$v0 := -52.500$$

```
    Sodium current
```

$$m0 := 0.028$$

$$h0 := 0.998$$

```
    Sustained Potassium current
```

$$NA0 := 0.017$$

$$NB0 := 0.015$$

```
    Transient Potassium current
```

$$a0 := 0.055$$

$$b0 := 0.057$$

```
Current equations
    Sodium current
    Sustained Potassium current
    Transient Potassium current
    Leak current
    Total ionic current
```

$$INa := 7.000\ (\mathrm{v}(t) - 35)\ \mathrm{m}(t)^3\ \mathrm{h}(t)$$

$$IK := (\mathrm{v}(t) + 67)\ (1.440\ \mathrm{NA}(t)^2 + 2.880\ \mathrm{NB}(t))$$

$$IA := 12\ (\mathrm{v}(t) + 67)\ \mathrm{a}(t)^4\ \mathrm{b}(t)$$

$$ILeak := 0.020\ \mathrm{v}(t) + 0.400$$

$$ITotal := 7.000\ (\mathrm{v}(t) - 35)\ \mathrm{m}(t)^3\ \mathrm{h}(t) + (\mathrm{v}(t) + 67)\ (1.440\ \mathrm{NA}(t)^2 + 2.880\ \mathrm{NB}(t)) + 12\ (\mathrm{v}(t) + 67)\ \mathrm{a}(t)^4\ \mathrm{b}(t) + 0.020\ \mathrm{v}(t) + 0.400$$

```
Equilibrium current as function of voltage
    Sodium current
    Sustained Potassium current
    Transient Potassium current
    Leak current
```

$$\frac{7.000\ (\mathrm{v}(t) - 35)\ \mathrm{h}(t)}{\left(1 + \mathbf{e}^{\left(-3 - \frac{t}{8}\right)}\right)^3}$$

$$\frac{7.000\,(\mathrm{v}(t)-35)}{\left(1+\mathbf{e}^{\left(-3-\frac{t}{8}\right)}\right)^3\,(1+\mathbf{e}^{(7.632+0.263\,t)})}$$

$$INainf := \frac{7.000\,(t-35)}{\left(1+\mathbf{e}^{\left(-3-\frac{t}{8}\right)}\right)^3\,(1+\mathbf{e}^{(7.632+0.263\,t)})}$$

$$(\mathrm{v}(t)+67)\left(\frac{1.440}{(1+\mathbf{e}^{(0.898-0.060\,t)})^2}+2.880\,\mathrm{NB}(t)\right)$$

$$(\mathrm{v}(t)+67)\left(\frac{1.440}{(1+\mathbf{e}^{(0.898-0.060\,t)})^2}+\frac{2.880}{1+\mathbf{e}^{(0.589-0.068\,t)}}\right)$$

$$IKinf := (t+67)\left(\frac{1.440}{(1+\mathbf{e}^{(0.898-0.060\,t)})^2}+\frac{2.880}{1+\mathbf{e}^{(0.589-0.068\,t)}}\right)$$

$$\frac{12\,(\mathrm{v}(t)+67)\,\mathrm{b}(t)}{(1+\mathbf{e}^{(-0.879-0.071\,t)})^4}$$

$$\frac{12\,(\mathrm{v}(t)+67)}{(1+\mathbf{e}^{(-0.879-0.071\,t)})^4\,(1+\mathbf{e}^{(0.152\,t+10.758)})}$$

$$IAinf := \frac{12\,(t+67)}{(1+\mathbf{e}^{(-0.879-0.071\,t)})^4\,(1+\mathbf{e}^{(0.152\,t+10.758)})}$$

$$ILeakinf := 0.020\,t+0.400$$

Total Equilibrium current

$$ITotalinf := \frac{7.000\,(t-35)}{\left(1+\mathbf{e}^{\left(-3-\frac{t}{8}\right)}\right)^3\,(1+\mathbf{e}^{(7.632+0.263\,t)})} + (t+67)\left(\frac{1.440}{(1+\mathbf{e}^{(0.898-0.060\,t)})^2}+\frac{2.880}{1+\mathbf{e}^{(0.589-0.068\,t)}}\right) + \frac{12\,(t+67)}{(1+\mathbf{e}^{(-0.879-0.071\,t)})^4\,(1+\mathbf{e}^{(0.152\,t+10.758)})} + 0.020\,t+0.400$$

Plotting Time constants and equilibrium values

Sodium current

Sustained Potassium current

Transient Potassium current

Total Equilibrium current

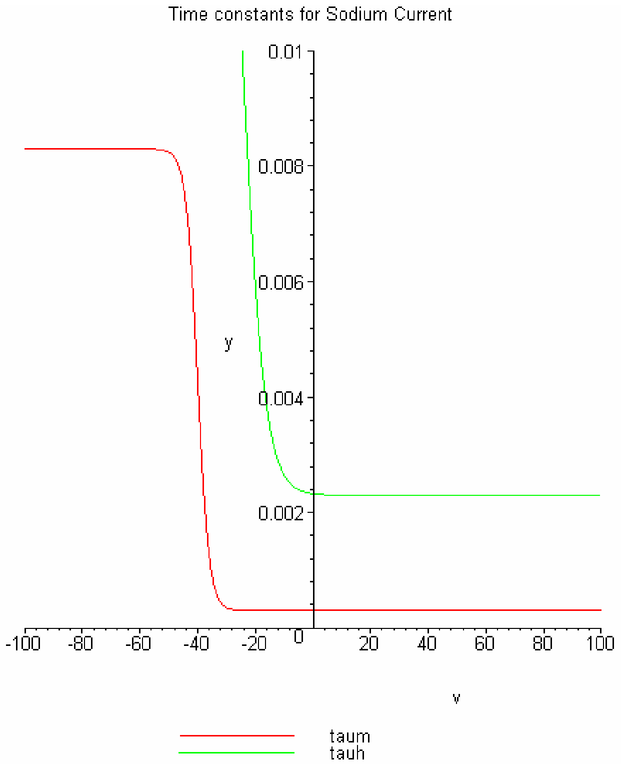
Time constants for Sodium Current
0.01
0.008
0.006
0.004
0.002
y
-100
-80
-60
-40
-20
0
20
40
60
80
100
v
taum
tauh

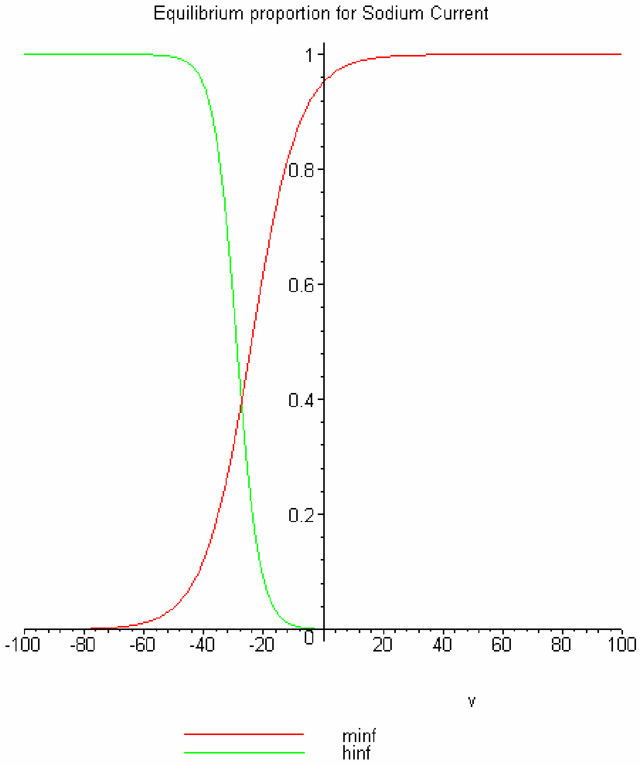
Equilibrium proportion for Sodium Current
1
0.8
0.6
0.4
0.2
-100
-80
-60
-40
-20
0
20
40
60
80
100
v
minf
hinf

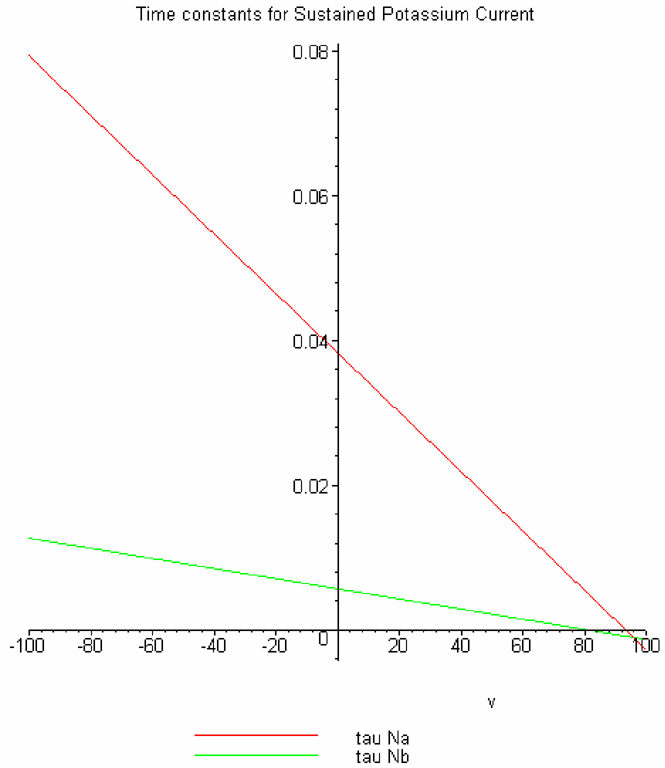
Time constants for Sustained Potassium Current
0.08
0.06
0.04
0.02
-100
-80
-60
-40
-20
0
20
40
60
80
100
v
tau Na
tau Nb

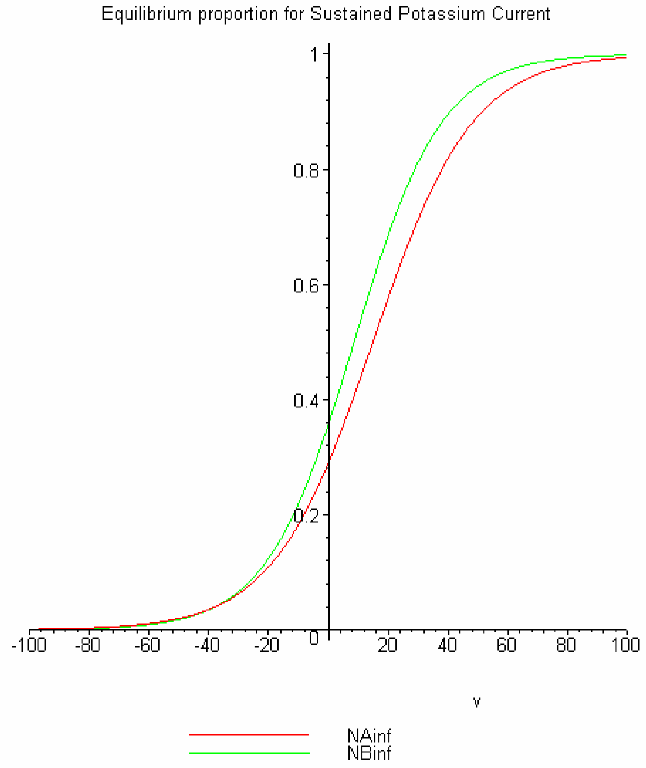
Equilibrium proportion for Sustained Potassium Current
1
0.8
0.6
0.4
0.2
-100
-80
-60
-40
-20
0
20
40
60
80
100
v
NAinf
NBinf

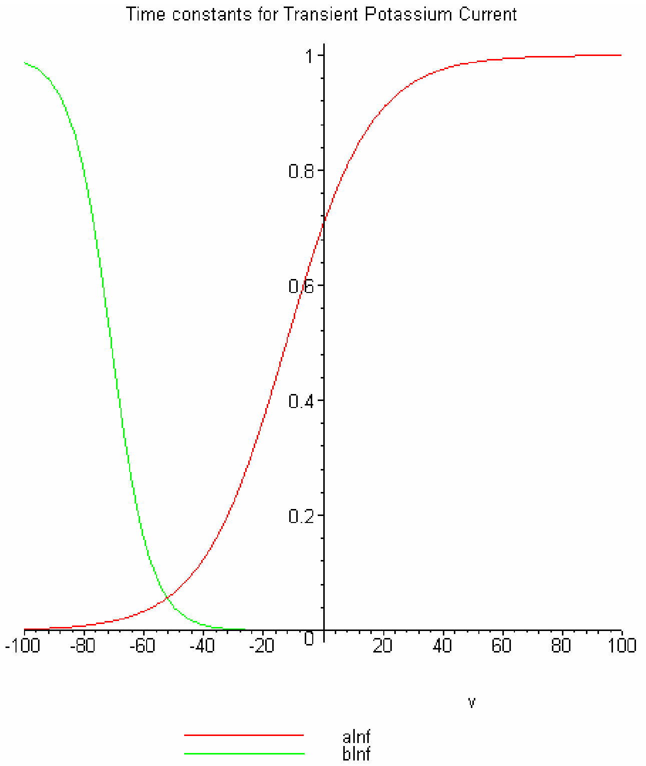
Time constants for Transient Potassium Current
1
0.8
0.6
0.4
0.2
0
-100
-80
-60
-40
-20
20
40
60
80
100
v
aInf
bInf

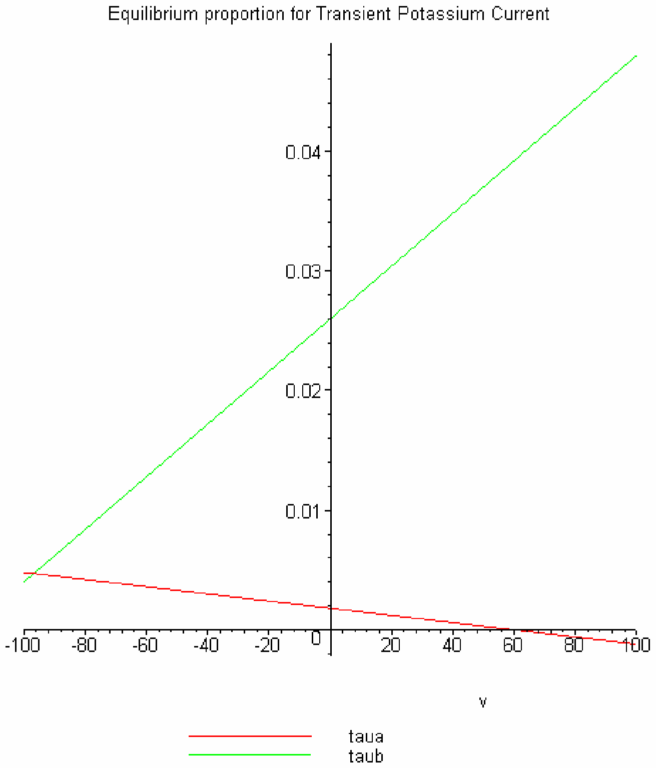
Equilibrium proportion for Transient Potassium Current
0.04
0.03
0.02
0.01
0
-100
-80
-60
-40
-20
20
40
60
80
100
v
taua
taub

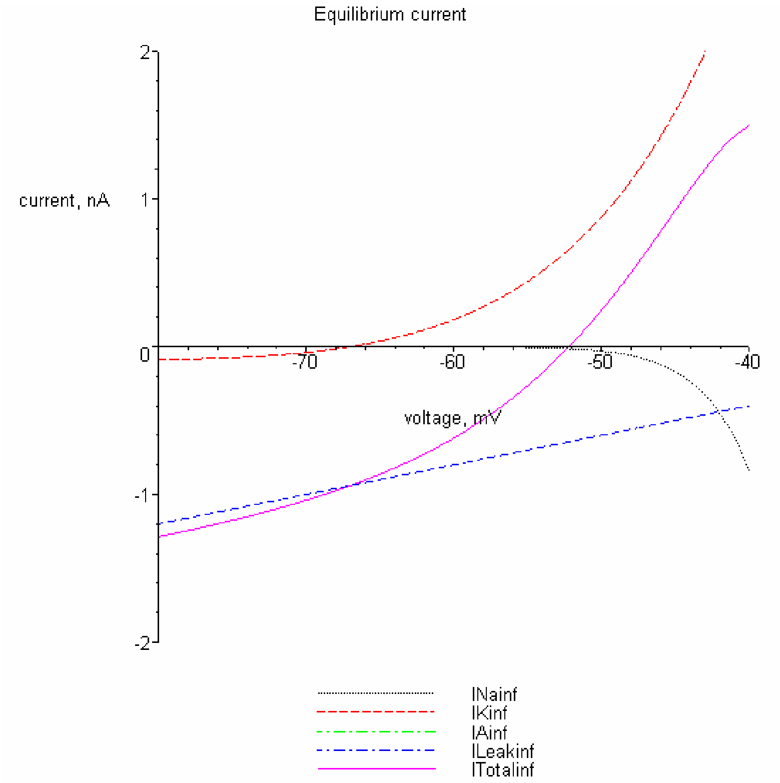
Equilibrium current
current, nA
voltage, mV
2
1
0
-1
-2
-70
-60
-50
-40
INainf
IKinf
IAinf
ILeakinf
ITotalinf
